# VvWRKY8 represses stilbene synthase genes through direct interaction with VvMYB14 to control resveratrol biosynthesis in grapevine

**DOI:** 10.1093/jxb/ery401

**Published:** 2018-11-16

**Authors:** Jinzhu Jiang, Huifen Xi, Zhanwu Dai, Fatma Lecourieux, Ling Yuan, Xianju Liu, Barunava Patra, Yongzan Wei, Shaohua Li, Lijun Wang

**Affiliations:** 1 Beijing Key Laboratory of Grape Sciences and Enology, CAS Key Laboratory of Plant Resources, Institute of Botany, Chinese Academy of Sciences, Beijing, China; 2 University of Chinese Academy of Sciences, Beijing, China; 3 EGFV, Bordeaux Sciences Agro, CNRS, INRA, ISVV, Université de Bordeaux, Villenave d’Ornon, France; 4 Department of Plant and Soil Sciences, University of Kentucky, Kentucky, USA

**Keywords:** Fine-tuning, grapevine, protein interaction, resveratrol, VvMYB14, VvWRKY8

## Abstract

Resveratrol (Res) is a stilbenoid, a group of plant phenolic metabolites derived from stilbene that possess activities against pests, pathogens, and abiotic stresses. Only a few species, including grapevine (*Vitis*), synthesize and accumulate Res. Although stilbene synthases (STSs) have been isolated and characterized in several species, the gene regulatory mechanisms underlying stilbene biosynthesis are still largely unknown. Here, we characterize a grapevine WRKY transcription factor, VvWRKY8, that regulates the Res biosynthetic pathway. Transient and stable overexpression of *VvWRKY8* in grapevine results in decreased expression of *VvSTS15/21* and *VvMYB14*, as well as in a reduction of Res accumulation. VvWRKY8 does not bind to or activate the promoters of *VvMYB14* and *VvSTS15/21*; however, it physically interacts with VvMYB14 proteins through their N-terminal domains to prevent them from binding to the *VvSTS15/21* promoter. Application of exogenous Res results in the stimulation of *VvWRKY8* expression and in a decrease of *VvMYB14* and *VvSTS15/21* expression in grapevine suspension cells, and in the activation of the *VvWRKY8* promoter in tobacco leaves. These results demonstrate that VvWRKY8 represses *VvSTS15/21* expression and Res biosynthesis through interaction with VvMYB14. In this context, the VvMYB14-VvSTS15/21-Res-VvWRKY8 regulatory loop may be an important mechanism for the fine-tuning of Res biosynthesis in grapevine.

## Teaser

VvWRKY8 interacts with the stilbene synthase transcriptional activator VvMYB14 to repress resveratol biosynthesis in grapevine.

## Introduction

Resveratrol (Res; 3,5,4′-trihydroxystilbene) is a stilbenoid, a class of plant-derived phenolic metabolites with activities against pests, pathogens, and abiotic stresses (Adrian *et al.*, 1997; Adrian and Jeandet, 2012). Res has been extensively studied for its antioxidant, immunomodulatory, anti-inflammatory, and anti-angiogenic effects, and for its potential roles in chemoprevention of cancer and cardioprotection (Kalantari and Das, 2010; Pangeni *et al.*, 2014; Weiskirchen and Weiskirchen, 2016). However, Res is naturally synthesized in only a few plant species. It was first isolated in 1939 from roots of white hellebore (*Veratrum grandiflorum*) (Takaoka, 1939), and then in 1964 from roots of Japanese knotweed (*Polygonum cuspidatum*) (Nonomura *et al.*, 1963). In 1974, it was characterized as a phytoalexin in the leaves of Vitaceae (Langcake and Pryce, 1976). The production of this phytoalexin is stimulated when Vitaceae are exposed to fungal infections, ozone, injury, wounding, and UV-C irradiation (Langcake and Pryce, 1976). Grapevine (*Vitis*) is currently the main source of supply of Res worldwide because of its extensive cultivation and high production efficiency (Weiskirchen and Weiskirchen, 2016). With an increasing demand for a natural source of Res, it is important to determine the gene regulatory pathway controlling its production.

Res is synthesized in the plant through the phenylalanine/polymalonate pathway, under the synergistic action of a series of related enzymes. Biosynthesis of Res and other phenylpropanoids begins with the synthesis of *trans*-cinnamic acid or its derivative *p*-coumaric acid from phenylalanine or tyrosine, respectively (Yu and Jez, 2008). Stilbene synthase (STS; EC2.3.1.95) catalyses the direct formation of Res from three malonyl-CoA units and one *p*-coumaroyl-CoA (Austin and Noel, 2003). Catalysed by glycosyltransferase (3-*O*-β-glycosyltransferases, 3-*O*-GT), Res is then converted into the corresponding glycoside (peceid, Pd). STS belongs to the type-III polyketide synthase enzyme superfamily and shares a high amino acid sequence identity with chalcone synthase (CHS; EC 2.3.1.74) (Austin and Noel, 2003). Vannozzi *et al.* (2012) identified 33 full-length sequences encoding *STS* genes in *Vitis vinifera*, which were clustered into three main groups designated as A, B, and C. Compared with those from groups A and C, *VvSTS* genes from group B are highly responsive to abiotic stresses, with wounding resulting in a 7- to 186-fold increase in transcription after 24 h. When grapevine leaf discs are exposed to UV-C light, their *VvSTS* transcription increases by 11- to 27-fold (Vannozzi *et al.*, 2012). However, the mechanisms controlling *VvSTS* gene expression remain unclear.

A limited number of transcription factors (TFs) regulating phenylpropanoid biosynthesis have been identified in a wide range of plant species. In grapevine, two MYB TFs, VvMYB14 and VvMYB15, trans-activate the promoters of *VvSTS29* and *VvSTS41* (Höll *et al*., 2013), and VvMYB14 directly binds to the *VvSTS48* promoter (Fang *et al.*, 2014). Very recently, Vannozzi *et al.* (2018) used *V. vinifera* suspension cell cultures to show that VvWRKY24 acts as a singular effector for *VvSTS29* promoter activation whereas VvWRKY3 acts through a combinatorial effect with VvMYB14 only through transient expression. In previous studies, we found that the expression of *VvSTS*s were largely up-regulated and Res concentration increased in leaves of *V. vinifera* after exposure to UV-C irradiation (Xi *et al.*, 2014, 2015). Interestingly, MYB and WRKY TFs such as VvMYB14, VvMYB15, and the previously uncharacterized *V. vinifera* WRKY 57-like (probe set ID: 1610775_s_at; GSVIVT01010525001) are up-regulated by 100- to 200-fold (Xi *et al.*, 2014). Using gene information from the PLEXdb database and Grape Genome Browser (The French–Italian Public Consortium for Grapevine Genome Characterization, 2007), we designated this WRKY TF as VvWRKY8 according to the VvWRKY phylogenetic tree (Wang *et al.*, 2014). WRKY TFs have been well characterized for their roles in regulating the production of valuable natural products such as phenylpropanoids, alkaloids, and terpenes by regulating metabolic pathway genes (Kato *et al.*, 2007; Guillaumie *et al.*, 2010; Singh *et al.*, 2017). Among the 59 *V. vinifera* WRKYs, VvWRKY2 is known to regulate lignin production (Guillaumie *et al.*, 2010), while VvWRKY26 is specific for the control of proanthocyanidin biosynthesis (Amato *et al*., 2016). We therefore speculated that VvWRKY8 may directly or indirectly regulate *VvSTS* expression.

In this study, we found that *VvWRKY8* is strongly co-expressed with *VvMYB14* and *VvSTS*s in grapevine leaves after UV-C treatment. Overexpression of *VvWRKY8* resulted in decreases of *VvSTS15/21* expression and Res accumulation in the leaves. Although VvWRKY8 does not specifically bind to or activate the promoters of *VvMYB14* or *VvSTS15/21*, it physically interacts with VvMYB14 through the N–terminal domains of both proteins. The VvWRKY8-VvMYB14 heterodimer prevents the VvMYB14 N-terminal DNA-binding domain from binding to the promoter region of *VvSTS15/21*. In addition, application of exogenous Res induces the expression of *VvWRKY8* and decreases the expression of *VvMYB14* and *VvSTS15/21* in grapevine suspension cells. Furthermore, it activated the *VvWRKY8* promoter in leaves of tobacco. These results suggest that VvWRKY8 negatively regulates *VvSTS15/21* by sequestrating its transcriptional activator, VvMYB14. A regulatory loop involving VvMYB14-VvSTS15/21-Res-VvWRKY8 may act as an important mechanism for the fine-tuning of Res biosynthesis in grapevine.

## Materials and methods

### Plant materials and growth conditions

Grapevine (*Vitis vinifera*), tobacco (*Nicotiana benthamiana*), and maize (*Zea mays*) were used in this study. Grapevines were grown in a vineyard at the Institute of Botany, Chinese Academy of Sciences, Beijing, China. Tobacco was planted in an illuminated chamber with a day/night cycle of 16/8 h light/dark at 25/20 °C. Maize plants were grown in a dark chamber at 25 °C, and at 10 d after sowing the second leaves were used to isolate protoplasts.

### UV-C irradiation of grapevine leaves

UV-C irradiation of grapevine leaves was performed as described by Xi *et al.* (2015). Mature (30-d-old), healthy leaves of similar size were detached from the shoots of cultivar ‘Hongbaladuo’, the leaf petioles were immediately inserted into water, and then transferred to triangular flasks containing double-deionized water (ddH_2_O). All leaves were incubated in the dark at 25 °C for 30 min, and then the leaf abaxial surfaces were exposed for 10 min to 6 W m^−2^ irradiation from a UV-C lamp (Model ZW30S26W, Beijing Lighting Research Institute, China). The leaves remained in the flasks in the dark until sampling. Control leaves were not irradiated. Samples were collected at 0, 3, 6, 12, 24, and 48 h after initiation of the treatment. All treated and control samples were replicated three times, and each replication consisted of six leaves.

### *VvWRKY8* gene isolation and analysis

Total RNA was extracted from mature leaves of *V. vinifera* cv. ‘Hongbaladuo’ using an E.Z.N.A.^®^ Plant RNA Kit (Omega Bio-tek, USA) according to the manufacturer’s instructions. Based on the gene sequence of *VvWRKY8* obtained from the Grape Genome Browser (http://www.genoscope.cns.fr/externe/GenomeBrowser/Vitis/), the primer pair for *VvWRKY8* was designed using Primer3Plus (http://www.primer3plus.com/cgi-bin/dev/primer3plus.cgi). *VvWRKY8* was cloned from cDNA by PCR (PrimeSTAR^®^ Max DNA Polymerase, Takara, China). The PCR products were ligated into the pLB simple vector (TIANGEN, China) and subsequently transformed into *Escherichia coli* TOP10. Positive colonies were selected and amplified, and then sequenced by Biomed Gene Technology Co., Ltd. The primers used for gene isolation are listed in Supplementary Table S1 at *JXB* online.

The deduced amino acid sequence of *VvWRKY8* was aligned with known homologous genes from *Artemisia annua*, *Arabidopsis thaliana*, and *Coptis japonica* (AaGSW1, AtWRKY75, and CjWRKY1, respectively) using Clustal X2 (Thompson *et al.*, 1997) with default settings. The alignment results were edited and marked using GeneDoc. The protein sequences used for alignment are shown in Supplementary Table S2.

### Gene expression analyses

Quantitative RT-PCR was conducted as described previously (Höll *et al*., 2013; Xi *et al.*, 2015) and the relative expression level of each gene was calculated using ΔΔ*C*_T_ (cycle threshold) method (Schefe *et al.*, 2006), with *VvActin7* (XM_002282480.4) as an internal control (Gutha *et al.*, 2010). Sucrose phosphate synthase 1 (*VvSPS1*), a gene involved in sucrose metabolism but not related to the Res biosynthesis pathway, was chosen as a negative control. The primer pairs used to detect *VvWRKY8*, *VvMYB14*, *VvSTS*s, and *VvSTS15/21* were designed using Primer3Plus. The primers used for qRT-PCR analyses are listed in Supplementary Table S1. The primer pair designed for *VvSTS*s could detect 25 *VvSTS*s, which included Group A members (*VvSTS1*, *VvSTS3*, *VvSTS5*, and *VvSTS6*) and Group B members (*VvSTS7*, *VvSTS8*, *VvSTS9*, *VvSTS10*, *VvSTS15*, *VvSTS21*, *VvSTS27*, *VvSTS29*, *VvSTS31*, *VvSTS33*, *VvSTS35*, *VvSTS37*, *VvSTS38*, *VvSTS39*, *VvSTS41*, *VvSTS42*, *VvSTS43*, *VvSTS45*, *VvSTS46*, *VvSTS47*, and *VvSTS48*) (Vannozzi *et al.*, 2012). All qRT-PCR analyses were performed with three independent biological replicates.

### Subcellular localization

For subcellular localization, the *VvWRKY8* coding sequence was amplified using a primer pair with a unique restriction site. The PCR product was then cloned in-frame into the pEZS-NL transient expression vector (pEZS-NL-VvWRKY8). Maize protoplasts were isolated and transfected according to the protocol described by Sheen *et al.* (1995), with minor modifications (Li *et al.*, 2012; Han *et al.*, 2015). After maize protoplasts were transfected with pEZS-NL-VvWRKY8, they were incubated in darkness overnight. They were then harvested by gentle centrifugation and stained with 0.1 g l^−1^ DAPI (Sigma-Aldrich) for 10 min. The VvWRKY8 localization pattern was determined by visualizing enhanced green fluorescent protein (eGFP) fluorescence using a Leica TCS SP5 Confocal Scanning Microscope, and the nuclei were visualized by DAPI ﬂuorescence. The peak excitation wavelength of eGFP and DAPI were 488 nm and 408 nm, respectively.

### Yeast one-hybrid assays

Yeast one-hybrid (Y1H) assays were performed using the Matchmaker One-Hybrid System (Clontech, USA) according to the manufacturer’s instructions. Full-length coding sequences of *VvMYB14* or *VvWRKY8* were subcloned in-frame into the pGAD424 vector (AD-VvMYB14 or AD-VvWRKY8), respectively. The promoters of *VvMYB14* or *VvWRKY8* (*proVvMYB14* or *proVvWRKY8*), and the common promoter fragment of *VvSTS15* and *VvSTS21* (*proVvSTS15/21*) were cloned into the pLacZi vector, respectively. The AD-fusion effectors were co-transformed with the *LacZ* reporters into yeast strain EGY48, and the transformants were selected and grown on synthetically defined (SD)/–Trp/–Ura selection media. The selected transformants were further grown on SD/–Trp/–Ura selection media supplied with 80 mg l^−1^ 5-bromo-4-chloro-3-indolyl-β-D-galactopyranoside (X-Gal) for color development. For the AD-VvMYB14, VvWRKY8, and *proVvSTS15/21* co-transformed experiment, *VvWRKY8* was subcloned into the pGADT7 vector, and the transformants were selected and grown on SD/–Trp/–Leu/–Ura selection media. The transformants were further grown on SD/–Trp/–Leu/–Ura selection media supplied with 80 mg l^−1^ X-Gal for color development. The primers used for the Y1H assays are listed in Supplementary Table S1.

### Plasmid construction for plant transformation

For plant transformation, the full-length coding sequences of *VvMYB14*, *VvWRKY8*, or *GUS* were ampliﬁed using the corresponding gene-specific primer pairs (Supplementary Table S1). The PCR products were then recombined into the pDONR221-P1P2, P1P4, and P3P2 entry vectors by Gateway BP recombination reactions (Life Technology, USA). *GUS*, *VvMYB14*, and *VvWRKY8* were then recombined into the pBiFC-2in1-CC vector (Grefen and Blatt, 2012) and the pH7WG2D vector (Karimi *et al.*, 2002) by Gateway LR recombination reactions. We obtained a series of recombinant vectors named as pBiFC-2in1-CC-GUS-GUS, pBiFC-2in1-CC-VvMYB14-GUS, pBiFC-2in1-CC-GUS-VvWRKY8, pBiFC-2in1-CC-VvMYB14-VvWRKY8, pH7WG2D-GUS, pH7WG2D-VvMYB14, and pH7WG2D-VvWRKY8.

### Transient luciferase (LUC) expression assays

The promoters of *VvMYB14* or *VvWRKY8* (*proVvMYB14* or *proVvWRKY8*), and the common promoter fragment of *VvSTS15* and *VvSTS21* (*proVvSTS15/21*) were respectively cloned into the pGreenII 0800-LUC vector (Hellens *et al.*, 2005). The vectors pBiFC-2in1-CC-GUS-GUS, pBiFC-2in1-CC-VvMYB14-GUS, pBiFC-2in1-CC-GUS-VvWRKY8, and pBiFC-2in1-CC-VvMYB14-VvWRKY8 were transfected into maize protoplasts with the *proVvSTS15/21::LUC* reporter vector. The protoplasts transfected with vectors were pelleted and resuspended in luciferase cell culture lysis reagent (Promega, USA) after incubation in darkness overnight. The vectors pH7WG2D-GUS, pH7WG2D-VvMYB14, and pH7WG2D-VvWRKY8 were transformed into tobacco leaves with corresponding promoter-*LUC* reporter vectors. Tobacco leaves were harvested 3 d after *Agrobacterium*-mediated transformation and 0.1-g samples of powdered tissue were used for extracting total proteins. Activities of firefly LUC and renilla LUC were measured using a GloMax 20/20 luminometer (Promega, USA) according to the manufacturer’s instructions. The relative activity was expressed as the ratio of firefly LUC/renilla LUC (Höll *et al*., 2013). The primers used for transient LUC expression assays are listed in Supplementary Table S1.

### Yeast two-hybrid assays

Yeast two-hybrid (Y2H) assays were performed using the Matchmaker Gold Yeast Two-Hybrid System (Clontech, USA). According to the manufacturer’s instructions, the full-length, C-terminus-deleted or N-terminus-deleted coding sequences of *VvMYB14* and *VvWRKY8* were subcloned in-frame into the pGADT7 vector and pGBKT7 vector, respectively. Different combinations of pGADT7 and pGBKT7 recombinant vectors were co-transformed into yeast strain Y2HGold and the transformants were grown on SD/–Leu/–Trp selection media. Positive colonies were plated onto SD/–Leu/–Trp/–His/–Ade selection media supplied with 40 mg l^−1^ 5-bromo-4-chloro-3-indolyl-α-D-galactopyranoside (X-α-Gal) to test for possible interactions. Combinations of AD-T with BD-p53 and BD-Lam served as positive and negative controls, respectively. For two-hybrid library screening, the prey cDNA library of *V. vinifera.* cv. ‘Pinot Noir’ was constructed according to the user manual of Make Your Own ‘Mate & Plate TM’ Library System (Clontech, USA). The positive strains were selected on SD/–Leu/–Trp/–Ade/–His selection media supplied with 40 mg l^−1^ X-α-Gal and 200 μg l^−1^ Aureobasidin A (AbA). The primers used for the Y2H assays are listed in Supplementary Table S1.

### Fluorescence resonance energy transfer-acceptor photobleaching (FRET-AB) assays and bimolecular fluorescence complementation (BiFC) assays

To generate the FRET and BiFC constructs, *GUS*, *VvMYB14*, and *VvWRKY8* were recombined into the pFRETtv-2in1-NN vector (Hecker *et al.*, 2015) and the pBiFC-2in1-NN vector (Grefen and Blatt, 2012) through corresponding Gateway entry vectors. We obtained the recombinant vectors named as pFRETtv-2in1-VvMYB14-mTRQ2/VvWRKY8-mVenus, pBiFC-2in1-NN-GUS/VvWRKY8, and pBiFC-2in1-NN-VvMYB14/VvWRKY8. The fusion proteins were transiently expressed in tobacco leaves by agro-infiltration. The chimeric fluorescence of the fusion proteins was detected 3 d after infiltration. For FRET-AB assays, the fluorescence images were acquired using an Olympus FV1000MPE Multiphoton Laser Scanning Microscope system. The peak excitation wavelength of mTRQ2 and mVenus were 458 nm and 488 nm, respectively. AB was performed using a bleaching routine with the 488-nm laser (mVenus) line at 100% intensity and 15 frames. For BiFC assays, the fluorescence images were acquired using a Leica TCS SP5 Confocal Scanning Microscope system. The peak excitation wavelength of yellow fluorescent protein (YFP) and red fluorescent protein (RFP) were 488 nm and 543 nm, respectively. RFP fluorescence was a marker of transformation efficiency.

### Transient and stable transformation of *VvWRKY8* in grapevine

Transient transformation of grapevine leaves was conducted according to a protocol described by Xu *et al*. (2010). *Agrobacterium tumefaciens* strain GV3101, harbouring pH7WG2D, pH7WG2D-VvWRKY8, was cultured at 28 °C in Luria Bertani (LB) liquid media with 50 mg l^−1^ rifampin, 50 mg l^−1^ gentamicin, and 100 mg l^−1^ spectinomycin. When the optical density at 600 nm (OD_600_) of the culture reached ~1.0, *Agrobacterium* cells were harvested and resuspended in induction buffer [10 mM MgCl_2_, 10 mM MES, pH 5.6, 2% (w/v) sucrose, and 150 µM acetosyringone], and the OD_600_ was adjusted to 0.6. The resuspended *Agrobacterium* cells were then incubated for 3 h at 28 °C before being used for inﬁltration. Leaves of *Vitis amurensis* of approximately identical size were selected and immersed in the *Agrobacterium* suspension. The vacuum in the container was kept at 0.085 MPa until the whole leaves became hygrophanous, and was then slowly released. The infiltrated leaves were put in a preservative film-sealed tray, and the petioles were kept wet. The infiltrated leaves were maintained for 3 d under normal growth conditions (25^o^C, 16/8 light/dark), then washed with ddH_2_O three times, frozen immediately in liquid nitrogen, and kept at –80 °C until further use.

Stable transformation of grapevine was conducted according to a protocol described by Zhou *et al.* (2014). The *A. tumefaciens* strain GV3101, harbouring pH7WG2D, pH7WG2D-VvWRKY8 was used for the transformation. Then VvWRKY8 was transformed into somatic embryos of *V. vinifera*. cv. ‘Thompson Seedless’. After regeneration and differentiation, the transgenic grapevine lines were planted in pots containing a mixed substrate (peat:perlite, 1:1) and grown in a controlled environment (25/18 °C day/night) for 90 d. Then the above-ground organs were harvested for measurement of Res and analysis of *VvSTS*s, *VvSTS15/21*, *VvMYB14*, and *VvWRKY8* expression.

### Extraction and determination of total Res

For Res extraction, tissue or callus samples were flash-frozen in liquid nitrogen and ground to a powder. Briefly, 1 g of tissue or callus was extracted with 15 ml extraction solution (methanol:ethyl acetate, 1:1 v/v) for 24 h at room temperature in the dark. After centrifugation at 20000 *g* at 4 °C for 10 min, the supernatant was evaporated at 40 °C until the solvent was volatilized completely and then dissolved in 2 ml methanol. The extract was filtered through a 0.45-μm PTFE membrane before HPLC analysis. All samples were analysed using a Waters Alliance® HPLC System (Waters e2695, Waters, USA) and a photodiode array (PDA, Waters 2998, Waters, USA) as described by Xi *et al.* (2015). *cis*-isomers (*cis*-Res and *cis*-Pd) and *trans*-isomers (*trans*-Res and *trans*-Pd) were detected at 288 nm and 306 nm, respectively, and PDA spectra were recorded from 240 nm to 600 nm. Known standards were run to identify elution times and mass fragments.

### Exogenous Res treatment

We conducted two experiments. Firstly, *trans*-Res was added into culture media of grapevine ‘41B’ cell suspension (*V. vinifera* cv. ‘Chasselas’×*V. berlandieri*) to 1 g l^−1^. After 6 h of treatment, the cells were collected and washed twice with ddH_2_O, then flash-frozen in liquid nitrogen, and stored at –80 °C until further use. Secondly, the *proVvWRKY8::LUC* reporter vector was transformed into tobacco leaves. After 2 d of treatment, the leaves were sprayed with 0.1 g l^−1^*trans*-Res, then maintained under darkness for 1 d. The leaf samples were washed with ddH_2_O, flash-frozen in liquid nitrogen, and stored at –80 °C until further use. Each of these experiments were performed with three independent biological replicates.

### Proteasome inhibition treatment of tobacco leaves

Tobacco leaves were transiently transformed with the VvWRKY8-6×His vector, then injected with 50 μM the proteasome inhibitor MG132 (Selleck, USA) or DMSO after 2 d of treatment. Total protein was extracted after 24 h incubation for immunoblot analysis of the VvWRKY8-6×His proteins using anti-His-tag antibody (Mei5 Biotech, China).

## Results

### Expression of *VvSTS*s, *VvWRKY8*, and *VvMYB14* in grapevine leaves in response to UV-C

Our previous studies suggested that VvWRKY8 may play a role in the regulation of Res synthesis (Xi *et al.*, 2014, 2015). We therefore analysed the temporal expression patterns of *VvSTS*s, *VvWRKY8*, and *VvMYB14* in grapevine leaves in response to UV-C. We designed a primer pair in the conserved region of the 25 *STS* mRNA sequences as described in our previous study (Xi *et al.*, 2015), and used the term *VvSTS*s to name them. The use of these primers may reflect the overall expression of *VvSTS* family members from groups A and B. Expression of *VvSTS*s increased sharply to peak at 12 h after UV-C irradiation, and then continuously decreased to return to the basal level at 48 h (Supplementary Fig. S1). After UV-C treatment, the *VvWRKY8* expression profile was similar to that of *VvSTS*s. In contrast to *VvSTS*s and *VvWRKY8*, the expression of *VvMYB14* exhibited a more rapid increase, peaking at 6 h, before gradually decreasing until 48 h (Supplementary Fig. S1). In the controls, expression of *VvSTS*s, *VvWRKY8*, and *VvMYB14* remained unchanged. These results further indicated that *VvWRKY8* could regulate Res biosynthesis with a different mechanism of action from *VvMYB14*.

### Cloning and sequence analysis of *VvWRKY8*

**Fig. 1.**
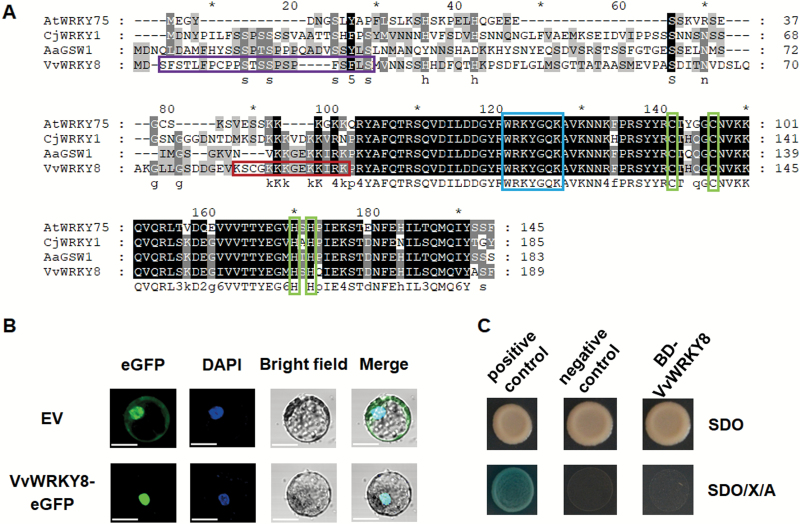
Sequence analysis, subcellular localization, and transcriptional activity of VvWRKY8. (A) The deduced amino acid sequence of VvWRKY8 aligned with its known homologs *Artemisia annua* AaGSW1, *Arabidopsis thaliana* AtWRKY75, and *Coptis japonica* CjWRKY1. Black and light gray shading indicate identical and similar amino acid residues, respectively. VvWRKY8 phosphorylation sites are shown by a purple box, the nuclear localization signal by a red box, the WRKY signature motif by a blue box, and the characteristic zinc finger motifs by green boxes. (B) Subcellular localization of VvWRKY8 in maize protoplasts. VvWRKY8 fused with enhanced green fluorescent protein (eGFP) was transfected into maize protoplasts and 0.1 g l^−1^ DAPI was then used to stain the protoplasts before visualization. eGFP fluorescence (green) and DAPI fluorescence (blue) observed using a confocal microscope are shown. Scale bars are 20 μm. (C) Analysis of VvWRKY8 transcriptional activity in yeast. Yeast cells expressing VvWRKY8 fused with yeast Gal4 binding domain (BD) were spotted on SD/–Trp (SDO) and SD/–Trp/X-α-Gal/AbA (SDO/X/A) selection media. Combinations of AD-T with BD-p53 and BD-Lam were used as positive and negative controls, respectively.

The full-length coding sequence of *VvWRKY8* was amplified by PCR from RNA isolated from leaves of grapevine cv. ‘Hongbaladuo’. Sequence analysis revealed that *VvWRKY8* contained a 570-bp ORF encoding a protein (XP_002275576.1) of 189 amino acids with a calculated molecular mass of 21.26 kDa and a predicted pI of 9.13. VvWRKY8 contained one putative WRKY domain (WRKYGQK) and one unique zinc finger-like motif (C–X_4_–C–X_23_–H–X–H) in its C-terminal region (Fig. 1A). VvWRKY8 belonged to the group IIc of the WRKY TF family according to the classification described by Eulgem *et al.* (2000). *In silico* analysis of the VvWRKY8 sequence using a prediction program (SMART, http://smart.embl-heidelberg.de/) indicated that it had two phosphorylation sites (^3^SFSTLFPCPPSTSSP***S***PF***S***FLS^24^) and a nuclear localization signal (^83^KSCGKKKGEKKIRK^96^) (Fig. 1A). We conducted phylogenetic analysis and multiple sequence alignment with other characterized WRKYs that are involved in the regulation of plant specialized metabolism and stress tolerance. The results showed that VvWRKY8 shared the highest sequence identity with AaGSW1 (55.56%), followed by CjWRKY1 (51.52%), and AtWRKY75 (45.45%) (Fig. 1A, Supplementary Fig. S2).

### VvWRKY8 is a nuclear localized protein lacking transcriptional activity

To determine the subcellular localization of VvWRKY8, a *VvWRKY8-GFP* fusion construct was transfected into maize protoplasts. Fluorescence analysis of protoplasts overexpressing *VvWRKY8-GFP* clearly indicated nuclear localization, in contrast to the GFP control fluorescence that was distributed uniformly throughout the cell (Fig. 1B).

To determine whether VvWRKY8 possesses transcriptional activity, *VvWRKY8* was fused in-frame to the Gal4 DNA-binding domain (BD) in the pGBKT7 vector. The resulting plasmid pGBKT7-VvWRKY8 and empty vector pGBKT7 were transformed individually into yeast strain Y2HGold. The yeast clones harbouring combinations of AD-T with BD-p53 and BD-Lam served as positive and negative controls, respectively. As shown in Fig. 1C, the positive control grew on SD/–Trp (SDO) and SD/–Trp/X-α-Gal/AbA (SDO/X/A) selection media, and exhibited alpha-galactosidase activity. In contrast, yeast cells harbouring pGBKT7-VvWRKY8 and the negative control only grew on SD/–Trp selection media, indicating that VvWRKY8 does not possess any transcriptional activity in yeast.

### Transient overexpression of *VvWRKY8* reduces Res concentration in grapevine leaves

**Fig. 2.**
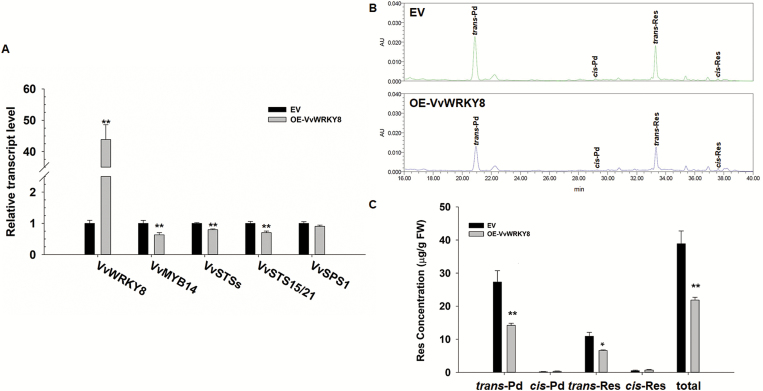
Transient overexpression of *VvWRKY8* and its effects on resveratrol (Res) concentration in grapevine leaves. Leaves of *Vitis amurensis* were infiltrated with *Agrobacterium* harbouring the pH7WG2D control and pH7WG2D-*VvWRKY8* overexpression constructs, and were sampled after 72 h. (A) qRT-PCR analysis of *VvWRKY8*, *VvSTS*s, and *VvSTS15/21* expression in leaves of *VvWRKY8*-overexpressing (OE) and control (empty vector, EV) plants. *VvSPS1* (sucrose phosphate synthase 1) was used as the negative control. Expression levels of genes were normalized to *VvActin7* and are represented as expression relative to the EV value, which was set to 1. (B, C) Res was extracted from EV and *VvWRKY8*-OE leaves, then quantified by HPLC analysis. (B) Chromatograms of Res in EV and *VvWRKY8*-OE leaves. AU, relative abundance in arbitrary units. (C) Res concentration in EV and *VvWRKY8*-OE leaves. The concentrations were calculated according to the characteristic peak area. *trans*-Res, *trans*-resveratrol; *cis*-Res, *cis*-resveratrol; *trans*-Pd, *trans*-piceid; *cis*-Pd, *cis*-piceid. Data are means (±SE) from four independent replicates. Significant differences were determined using Student’s t-test: *P<0.05; **P<0.01.

To investigate how VvWRKY8 regulates Res biosynthesis in grapevine, an agro-infiltration transient assay of *VvWRKY8* was conducted in grapevine leaves. The expression of *VvWRKY8* under the control of the *CaMV 35S* promoter had increased ~43-fold at 2 d post-infiltration (Fig. 2A). Because the expression of *VvSTS15* and *VvSTS21* in group B are strongly up-regulated (145–850-fold) in grapevine leaves exposed to UV-C (Vannozzi *et al.*, 2012), we investigated their expression. Based on a high degree of similarity between the sequences, we designed the primers STS15/21-F/R to quantify the combined expression levels of *VvSTS15* and *VvSTS21* (*VvSTS15/21*). Surprisingly, the subsequent analysis showed that expression of *VvSTS*s and *VvSTS15/21* were down-regulated (~20% and ~30%, respectively). Moreover, *VvMYB14* expression also decreased (Fig. 2A). In parallel, we determined the concentrations of *trans*-Res, *cis*-Res, *trans*-Pd, and *cis*-Pd in infiltrated leaves. *VvWRKY8* overexpression led to a significant reduction of total Res concentration compared to control leaves infiltrated with the empty vector (EV) (Fig. 2B, C). In particular, *trans*-Pd and *trans*-Res exhibited significant reductions in *VvWRKY8*-overexpression leaves in comparison to control (EV) leaves (Fig. 2C).

### VvWRKY8 does not bind to or activate the promoters of *VvSTS15/21* and *VvMYB14*

The negative effects of VvWRKY8 on Res biosynthesis prompted us to test the possibility that VvWRKY8 inhibits *VvSTS* expression by binding to the promoter of *VvSTS* or *VvMYB14*. We cloned the common promoters (*proVvSTS15/21*) of *VvSTS15* and *VvSTS21* on the basis of their high sequence similarity (Supplementary Fig. S3), and the promoter of *VvMYB14*. To determine whether VvWRKY8 binds to these promoters, the full-length coding sequence of *VvWRKY8* fused with the B42 activation domain was cloned into the pGAD424 effector vector for Y1H assays. *proVvSTS15/21* was cloned into the pLacZi reporter vector. The VvWRKY8-AD effector vector was co-transformed with the *proVvSTS15/21::LacZ* reporter vector into yeast strain EGY48. The transformants were grown on SD/–Trp/–Ura selection media supplied with 80 mg l^−1^ X-Gal for color development. The results showed that the yeast cells harbouring VvWRKY8-AD/*proVvSTS15/21::LacZ* did not turn blue, whereas the positive control yeast cells harbouring VvMYB14-AD/*proVvSTS15/21::LacZ* did turn blue (Supplementary Fig. S4A). This suggested that VvWRKY8 does not bind to the *VvSTS15/21* promoter. Using a similar method, we also found that yeast cells harbouring VvWRKY8-AD/*proVvMYB14::LacZ* did not turn blue, suggesting that VvWRKY8 does not bind to *VvMYB14* promoter in yeast (Supplementary Fig. S4C).

We further investigated whether VvWRKY8 can activate the promoters of *VvSTS15/21* and *VvMYB14* in tobacco leaves. The full-length *VvWRKY8* coding sequence was sub-cloned into the pH7WG2D vector, and *proVvMYB14* and *proVvSTS15/21* were individually sub-cloned into the pGreenII 0800-LUC reporter vector. The VvWRKY8 effector and reporter combinations were transformed into tobacco leaves, and the activities of firefly LUC and renilla LUC were measured. We found that there was no difference in the ratio of firefly LUC/renilla LUC between *VvWRKY8*-overexpression and EV for *VvSTS15/21* or *VvMYB14* (Supplementary Fig. S4B, D). These results indicated that VvWRKY8 does not bind to or trans-activate the promoters of *VvSTS15/21* or *VvMYB14*, and confirmed the results obtained by Y1H assays.

We also used Y1H assays and transient luciferase expression assays to examine the possible binding and trans-activation ability of VvMYB14 on the *VvWRKY8* promoter. Similarly, we found that VvMYB14 does not bind to or trans-activate the *VvWRKY8* promoter (Supplementary Fig. S5A, B).

### VvWRKY8 physically interacts with VvMYB14

**Fig. 3.**
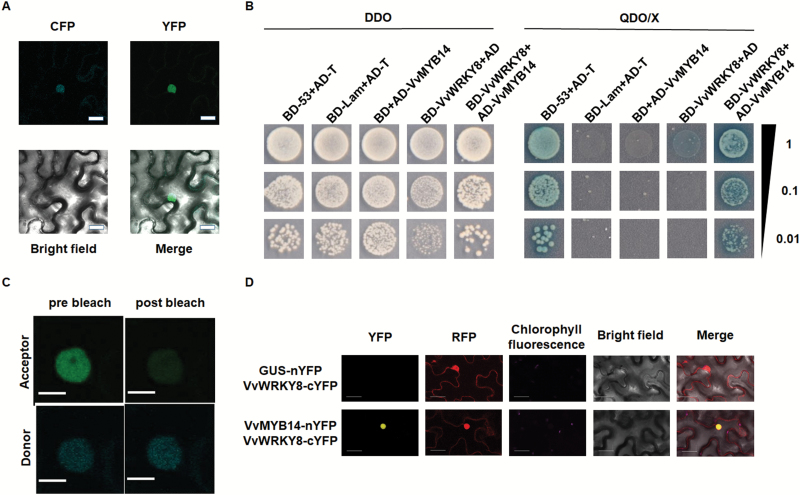
VvWRKY8 physically interacts with VvMYB14. (A) Subcellular co-localization of VvMYB14 and VvWRKY8. VvMYB14-CFP (cyan fluorescent protein) and VvWRKY8-YFP (yellow fluorescent protein) fusions were transformed into tobacco leaves and CFP fluorescence (cyan) and YFP fluorescence (green) were observed using a confocal microscope. Scale bars are 20 μm. (B) Yeast two-hybrid assays of the physical interaction of VvWRKY8 with VvMYB14. The protein interaction was examined using various combinations of prey and bait vectors. All transformants were conducted on SD/–Leu/–Trp (DDO) or SD/–Leu/–Trp/–His/–Ade/X-α-Gal (QDO/X) selection media. Interactions were determined on the basis of cell growth and cell color. Dilutions (1, 0.1, and 0.01) of saturated cultures were spotted on the plates. (C) Fluorescence resonance energy transfer-acceptor photobleaching (FRET-AB) assays of the interaction of VvWRKY8 with VvMYB14. The vector pFRETtv-2in1-VvMYB14-mTRQ2/VvWRKY8-mVenus was transformed into tobacco leaves, and acceptor (mVenus) and donor (mTRQ2) fluorescence were monitored prior to (pre bleach) and after (post bleach) bleaching. Scale bars are 10 mm. (D) bimolecular fluorescence complementation (BiFC) assays of the interaction of VvWRKY8 with VvMYB14. pBiFC-2in1-VvMYB14-nYFP/VvWRKY8-cYFP and pBiFC-2in1-GUS-nYFP/VvWRKY8-cYFP control vectors were transformed into tobacco leaves. YFP fluorescence (yellow), RFP fluorescence (red), and chlorophyll autofluorescence (purple) were observed by confocal microscopy. RFP fluorescence was used as a marker of transformation efficiency. Scale bars are 30 μm.

Since VvWRKY8 did not bind to or activate the promoters of *VvSTS15/21* or *VvMYB14*, we speculated that an interaction between the nucleus-localized VvWRKY8 and VvMYB14 (Fig. 3A) may affect Res biosynthesis. Yeast two-hybrid assays were used to test this hypothesis. The full-length coding sequences of *VvMYB14* and *VvWRKY8* were sub-cloned into the pGADT7 vector and pGBKT7 vector, respectively. Transformants harbouring AD-VvMYB14/BD-VvWRKY8 survived and appeared blue in SD/–Leu/–Trp/–His/–Ade selection media supplied with 40 mg l^−1^ X-α-Gal (Fig. 3B). This result suggested that VvWRKY8 interacts with VvMYB14 protein in yeast.

To further confirm the interaction between VvWRKY8 and VvMYB14, FRET-AB assays and BiFC assays were performed. The full-length coding sequences of *VvMYB14* and *VvWRKY8* were sub-cloned in-frame into pFRETtv-2in1-NN and pBiFC-2in1-NN vectors, and the *A. tumefaciens* strain harbouring these vectors was infiltrated into tobacco leaves. The chimeric fluorescence of the expressed fusion proteins was detected 3 d post infiltration. The FRET-AB assays showed an increase in fluorescence intensity of VvMYB14-mTRQ2 after bleaching VvWRKY8-mVenus (Fig. 3C), with FRET efficiency of 38.54%. In the BiFC assays, YFP fluorescence was detected in cells expressing VvMYB14-nYFP/VvWRKY8-cYFP, while no YFP fluorescence appeared in cells expressing GUS-nYFP/VvWRKY8-cYFP (Fig. 3D). These results further confirmed the presence of a direct interaction between VvWRKY8 and VvMYB14.

### VvWRKY8–VvMYB14 interaction mediated by the N-termini inhibits the binding of VvMYB14 to the *VvSTS15/21* promoter

**Fig. 4.**
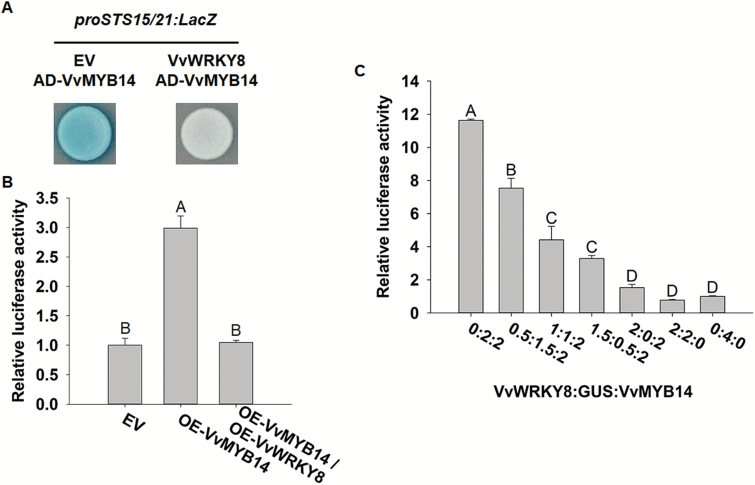
Effects of the interaction of VvWRKY8 with VvMYB14 on *proVvSTS15/21* activity. (A) Yeast one-hybrid assays. VvMYB14 was fused to the B42 activation domain (AD) and VvWRKY8 was overexpressed by the ADH1 promoter, and the resulting plasmids were co-transformed with the *proVvSTS15/21::LacZ* reporter into yeast cells. The transformants were further grown on SD/–Trp/–Leu/–Ura selection media supplied with 80 mg l^−1^ X-Gal for color development. (B) Transient expression assay of the *proVvSTS15/21::LUC* reporter with the VvMYB14 effector in the presence or absence of VvWRKY8 in maize protoplasts. The *proVvSTS15/21::LUC* reporter was co-transfected with the VvMYB14 effector and/or VvWRKY8 into maize protoplasts. EV represents the *proVvSTS15/21::LUC* reporter activity with the empty effector vector. Activities of the LUC protein were normalized to renilla LUC and are represented as activities relative to EV, which was set to 1. (C) Transient expression assay of the *proVvSTS15/21::LUC* reporter with the VvMYB14 transcriptional effector in the presence of increasing amounts of VvWRKY8 in tobacco leaves. The *proVvSTS15/21::LUC* reporter, VvMYB14 effector, and VvWRKY8 were co-transformed into tobacco leaves with an increasing ratio of *Agrobacterium* cells expressing *VvWRKY8* to those of *VvMYB14* (*VvWRKY8:VvMYB14 =* 0:2, 0.5:2, 1:2, 1.5:2, and 2:2). The control represented the reporter alone without any effectors. Activities of the LUC protein were normalized to renilla LUC and are represented as activities relative to the control, which was set to 1. Data are means (±SE) of three biological replicates. Different letters indicate significant differences according to Duncan’s test (*P*<0.01).

To explain how VvWRKY8 influences the expression of *VvSTS15/21* through interaction with VvMYB14, yeast EGY48 cells were co-transformed with VvWRKY8, AD-VvMYB14, and *proVvSTS15/21::LacZ*. Compared to EV controls, colonies harbouring VvWRKY8 showed a weaker blue intensity (Fig. 4A). In parallel, maize protoplasts were co-transfected with constructs overexpressing (OE) the two TFs (*VvWRKY8*-OE and *VvMYB14*-OE), individually or in combination, and the *proVvSTS15/21::LUC* reporter construct. Co-transfection with *VvWRKY8*-OE and *VvMYB14*-OE reduced luciferase relative activity when compared to single transfection with *VvMYB14*-OE (Fig. 4B). Competing interaction assays were performed in tobacco leaves by adding increasing amounts of *VvWRKY8* to a fixed amount of *VvMYB14*. VvMYB14-mediated activation of the *VvSTS15/21* promoter gradually decreased with increasing *VvWRKY8* content (Fig. 4C). These results suggested that the physical interaction between VvWRKY8 and VvMYB14 blocks VvMYB14 binding to the *VvSTS15/21* promoter in a dose-dependent manner.

**Fig. 5.**
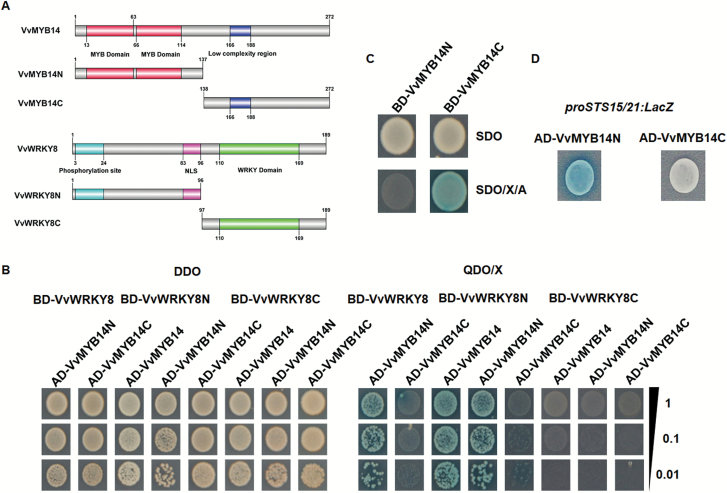
Interaction between amino (N)-terminal halves of VvWRKY8 and VvMYB14, and identification of the trans-activation and DNA binding termini of VvMYB14. (A) Diagram of the different constructs used for yeast two-hybrid assays. Both VvWRKY8 and VvMYB14 were divided into two halves, with the N- and C-terminal halves being designated N and C, respectively. The amino acid positions of these fragments are numbered. (B) Yeast two-hybrid assays. VvMYB14 and its halves were fused with the activation domain of Gal4, while VvWRKY8 and its halves were fused with the binding domain of Gal4. The protein interaction was examined using various combinations of prey and bait vectors. All transformants were conducted on SD/–Leu/–Trp (DDO) or SD/–Leu/–Trp/–His/–Ade/X-α-Gal (QDO/X) selection media. Interactions were determined on the basis of cell growth and cell color. Dilutions (1, 0.1, and 0.01) of saturated cultures were spotted on the plates. (C) Transcriptional activation tests for VvMYB14N and VvMYB14C in yeast. Yeast cells containing VvMYB14N and VvMYB14C protein fused with yeast Gal4 binding domain (BD) were spotted on SD/–Trp (SDO) and SD/–Trp/X-α-Gal/AbA (SDO/X/A) selection media. (D) Yeast one-hybrid assays. VvMYB14N and VvMYB14C were fused to the B42 activation domain (AD), and co-transformed with the *proVvSTS15/21::LacZ* reporter into yeast cells. The transformants were further grown on SD/–Trp/–Ura selection media supplied with 80 mg l^−1^ X-Gal for color development.

To identify the binding site within the heterodimer that was formed, the C-terminal and N-terminal halves of VvWRKY8 and VvMYB14 (designated as VvWRKY8C, VvWRKY8N, VvMYB14C, and VvMYB14N) were co-expressed in yeast (Fig. 5A). Yeast cells expressing VvWRKY8N/VvMYB14N, VvMYB14N/full-length VvWRKY8, and VvWRKY8N/full-length VvMYB14 appeared blue, indicating that the interaction was mediated by VvWRKY8N and VvMYB14N (Fig. 5B). In addition, we found that both BD-VvMYB14C and AD-VvMYB14N fusions were transcriptionally active in yeast (Fig. 5C, D), suggesting that the DNA binding domain of VvMYB14 is located at the N-terminus. This is consistent with previous reports showing that binding domains of MYB TFs usually reside at the N-terminus. Together, these results showed that the N-terminal interaction between VvWRKY8–VvMYB14 inhibits VvMYB14 binding and activation of the *VvSTS15/21* promoter.

### *VvWRKY8* overexpression down-regulates *VvSTS*s and reduces Res accumulation in stable transgenic grapevine calli and leaves

**Fig. 6.**
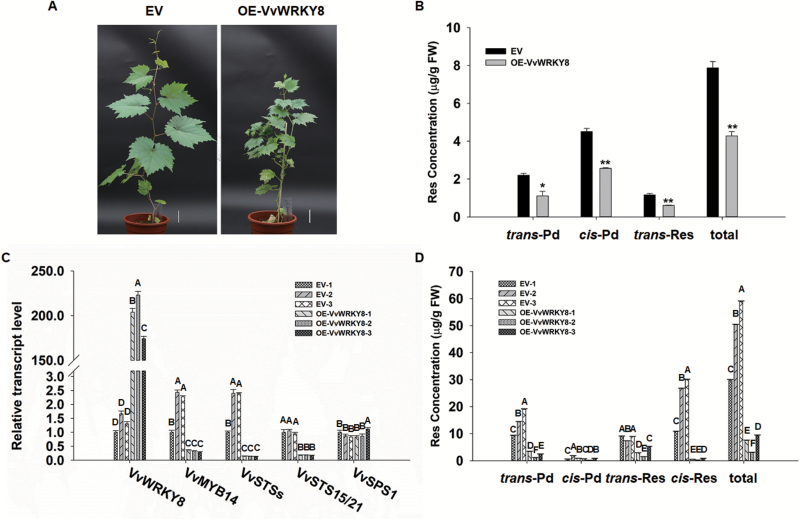
*VvWRKY8* overexpression decreases resveratrol (Res) concentration in grapevine calli and grapevine plants. VvWRKY8 was transformed to somatic embryos of *V. vinifera*. cv. ‘Thompson Seedless’ using the *Agrobacterium*-mediated method. After regeneration and differentiation, the transgenic grapevines were used for further experiments. (A) Phenotypes of empty-vector (EV) control and *VvWRKY8*-overexpressing (OE) grapevine. These grapevines were grown in feeding blocks for 90 d. Scale bars are 3 cm. (B) *trans*-Pd, *cis*-Pd, *trans*-Res, and total Res concentrations in EV and *VvWRKY8*-OE grapevine calli. Res was extracted from the EV and *VvWRKY8*-OE grapevine calli, then quantified by HPLC analysis. The concentrations were calculated according to the characteristic peak area. (C) qRT-PCR analysis of *VvWRKY8*, *VvSTS*s, and *VvSTS15/21* expression in grapevine leaves. *VvSPS1* was used as the negative control. Expression levels of genes were normalized to *VvActin7* and are represented as expression relative to EV-1, which was set to 1. (D) *trans*-Pd, *cis*-Pd, *trans*-Res, *cis*-Res, and total Res concentrations in EV and *VvWRKY8*-OE grapevine leaves. Res was extracted from the leaves of EV and *VvWRKY8*-OE transgenic grapevines, then quantified by HPLC analysis. The concentrations were calculated according to the characteristic peak area. Data are means (±SE) from three independent replicate. Significant differences between EV and *VvWRKY8*-OE in (B) were determined using Student’s *t*-test (*P<0.05; **P<0.01) and in (C, D) by Duncan’s test (indicated by different letters, *P*<0.05).

To further examine the function of VvWRKY8 in grapevine, *VvWRKY8* was transformed in *V. vinifera*. cv. ‘Thompson Seedless’ somatic embryos (Fig. 6A, Supplementary Fig. S6). We measured Res concentrations in control and transgenic calli, and found that in both no *cis*-Res was detectable; in contrast, *trans*-Pd, *cis*-Pd, and *trans*-Res concentrations were significantly reduced in transgenic calli when compared to control (Fig. 6B). Somatic embryos were then induced for grapevine plant development. Three *VvWRKY8*-OE and three EV control transgenic plant lines were obtained and used for further analysis. Compared with the controls, *VvWRKY8* expression was up-regulated by ~170-fold in overexpressing lines whereas expression of *VvSTS*s, *VvSTS15/21*, and *VvMYB14* was significantly down-regulated (Fig. 6C). The concentrations of *trans*-Pd, *trans*-Res, *cis*-Res, and total Res in *VvWRKY8*-OE transgenic lines were significantly lower than those of control lines (Fig. 6D).

### Res induces the expression of *VvWRKY8*

**Fig. 7.**
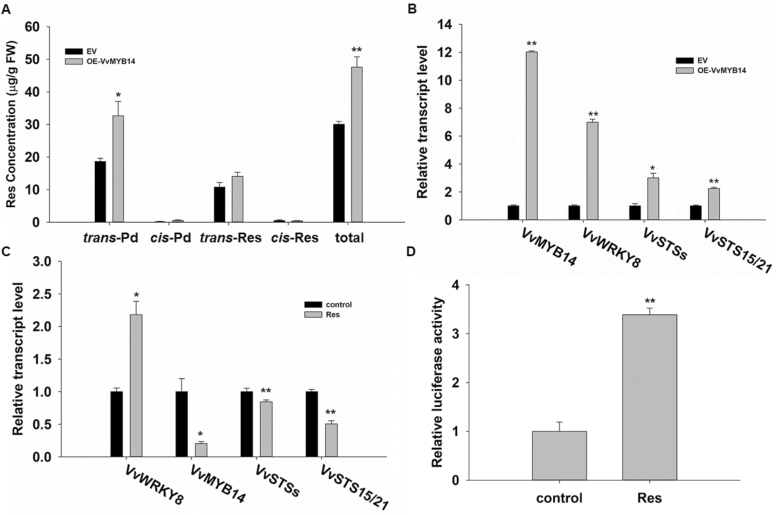
Resveratrol (Res) induces the expression of VvWRKY8. (A, B) Res accumulation and *VvWRKY8*, *VvMYB14*, *VvSTS*s, and *VvSTS15/21* expression in grapevine leaves transiently overexpressing *VvMYB14* (*VvMYB14*-OE) compared with the empty vector control (EV). Leaves of *Vitis amurensis* were infiltrated with *Agrobacterium* harbouring the pH7WG2D control and pH7WG2D-*VvMYB14* overexpression constructs, and were sampled after 72 h. (A) Res accumulation in *VvMYB14*-OE grapevine leaves. Res was extracted from EV and *VvMYB14*-OE leaves, then quantified by HPLC analysis. (B) Expression of *VvWRKY8*, *VvMYB14*, *VvSTS*s, and *VvSTS15/21* in EV and *VvMYB14*-OE grapevine leaves. Expression levels of genes were normalized to *VvActin7* and are represented as expression relative to EV, which was set to 1. (C) Expression of *VvMYB14*, *VvWRKY8*, *VvSTS*s, and *VvSTS15/21* in grapevine suspension cells after exogenous supply of *trans*-Res. Expression levels of genes were normalized to *VvActin7* and are represented as expression relative to the control, which was set to 1. (D) Transient expression assay of the *proVvWRKY8::LUC* reporter in tobacco leaves sprayed with exogenous *trans*-Res compared with the control. Activities of the LUC protein were normalized to renilla LUC and are represented as activities relative to the control, which was set to 1. Data are means (±SE) from three independent replicates. Significant differences were determined using Student’s *t*-test: *P<0.05; **P<0.01.

To further investigate the relationships among *VvWRKY8*, *VvMYB14*, and Res, *VvMYB14* was transiently overexpressed in grapevine leaves. We found that the total Res and *trans*-Pd concentrations increased by almost 100% (Fig. 7A), parallel to the expression level of *VvMYB14*, which was up-regulated by ~12-fold (Fig. 7B). Unexpectedly, we found that *VvWRKY8* expression was up-regulated by ~7-fold (Fig. 7B). This finding was puzzling as VvMYB14 did not bind to or activate the *VvWRKY8* promoter (Supplementary Fig. S5). To try to understand this result, we tested the possibility of feedback regulation between Res accumulation and *VvWRKY8* expression. Exogenous *trans*-Res was added to a grapevine cell suspension culture and we found that after 6 h of treatment the total Res concentration increased significantly (Supplementary Table S3), *VvWRKY8* expression increased by ~2-fold, whereas expression of *VvMYB14*, *VvSTS*s, and *VvSTS15/21* were significantly decreased (Fig. 7C). In addition, exogenous *trans*-Res was sprayed onto tobacco leaves that were transformed with *proVvWRKY8::LUC* and after 1 d of treatment the Res concentration had increased significantly (Supplementary Table S3). LUC activity controlled by *proVvWRKY8* in the treated leaves increased more than 3-fold compared with the control (Fig. 7D). These results indicated that *VvWRKY8* expression is induced by Res supply.

### VvWRKY8 is possibly regulated by the ubiquitin-proteasome pathway

TFs are known to be regulated at post-translational levels, and WRKY TFs are able to interact with multiple proteins (Chi *et al.*, 2013; Yang *et al.*, 2017*a*). We therefore searched for additional VvWRKY8 interacting partners by using it as a bait to screen a prey cDNA library from *V. vinifera.* cv. ‘Pinot Noir’. The full-length coding sequences of all positive clones were sub-cloned into the pGADT7 vector and their interaction with VvWRKY8 was verified by Y2H assays. A total of 17 candidates were confirmed (Supplementary Table S4). Among them, we identified three E3 ubiquitin-protein ligases that were homologous to MIEL1 (GSVIVT01033674001), RHB1A (GSVIVT01027002001), and LOG2 (GSVIVT01025674001), as well as one COP9 signalosome complex subunit 5b (GSVIVT01023827001) (Supplementary Fig. S7A). In addition, western blotting showed that when tobacco leaves transiently overexpressing *VvWRKY8* were treated with the proteasome inhibitor MG132, the amounts of VvWRKY8 protein increased significantly (Supplementary Fig. S7B). These results underlined a possible role for the ubiquitin ligase system in regulating VvWRKY8 activity and allowing the fine-tuning of Res biosynthesis.

## Discussion

For the last 20 years, the WRKY family has been widely known for its role in regulating abiotic and biotic stress tolerance in plants; however, accumulating evidence has indicated that WRKYs also regulate specialized metabolism such as the phenylpropanoid, alkaloid, and terpene pathways (Schluttenhofer and Yuan, 2015). For example, overexpression of four *Medicago truncatula* WRKY TFs in tobacco (*N. tabacum*) increases the levels of soluble and wall-bound phenolic compounds and of lignin (Naoumkina *et al.*, 2008). The disruption of stem-expressed WRKY genes in *M. truncatula* up-regulates TF genes such as the NAC factors NAM, ATAF1/2, and CUC2, as well as CCCH-type (C3H) zinc fingers (Wang *et al.*, 2010). Grapevine VvWRKY2 activates the *VvC4H* promoter in tobacco protoplasts (Guillaumie *et al.*, 2010). Based on our previous studies (Xi *et al.*, 2014, 2015), we investigated the temporal expression patterns of *VvWRKY8* and *VvSTS*s in grapevine leaves after UV-C treatment and found that they were highly correlated (Supplementary Fig. S1). *VvWRKY8* shares high sequence identity (51.52%) with *CjWRKY1* (Fig. 1A), which regulates benzylisoquinoline alkaloid biosynthesis in *C. japonica* (Kato *et al.*, 2007). VvWRKY8 was found to be localized in the nucleus (Fig. 1B), which is consistent with the putative transcriptional role of this protein. *VvWRKY8* overexpression resulted in a significant decrease in Res accumulation (Fig. 2), making VvWRKY8 the first negative regulator of Res biosynthesis to be characterized.

The involvement of WRKY TFs as positive or negative regulators of gene expression has been shown previously (Schluttenhofer and Yuan, 2015). Ishiguro and Nakamura (1994) identified the first WRKY TF (SWEET POTATO FACTOR1, SPF1) acting as a negative regulator of β-amylase expression in sweet potato. Most WRKY proteins (e.g. AtWRKY18, AtWRKY60, NtWRKY12, and GaWRKY1) have been shown to be transcriptional activators, whereas a few others (e.g. AtWRKY40, OsWRKY51, and OsWRKY71) act as repressors of transcription (Xu *et al.*, 2004; Xie *et al.*, 2006; van Verk *et al.*, 2008; Chen *et al.*, 2010). Overexpression of *OsWRKY13* up-regulates genes of the phenylpropanoid pathway in rice, while overexpression of *OsWRKY76* represses terpene and sakuranetin biosynthesis (Qiu *et al.*, 2008; Yokotani *et al.*, 2013). A VvWRKY8 homolog from *A. annua*, AaGSW1, positively regulates artemisinin and dihydroartemisinic acid concentrations by directly binding to the W-box motifs of the *CYP71AV1* and *AaORA* promoters (Chen *et al.*, 2017). Another VvWRKY8 homolog, CjWRKY1, increases the level of transcripts of all berberine biosynthetic genes (Kato *et al.*, 2007).

In the present study, VvWRKY8 showed no transcriptional activity in yeast (Fig. 1B). WRKY TFs lacking transcriptional activity have been observed in maize ZmWRKY17, chrysanthemum CmWRKY17, and wheat TaWRKY71-1 (Qin *et al.*, 2013; Li *et al.*, 2015; Cai *et al.*, 2017). In addition, Y1H assays showed that VvWRKY8 did not bind to the promoters of *VvSTS15/21* and *VvMYB14*. Furthermore, transient expression assays indicated that VvWRKY8 did not trans-activate the promoters of *VvSTS15/21* and *VvMYB14* (Supplementary Fig. S4). Taken together, these results led us to consider a possible mechanism for the repression of Res biosynthesis by VvWRKY8 through the disruption of the activity of some activators, such as VvMYB14. In this context, using Y2H, FRET-AB, and BiFC assays we confirmed the physical interaction between VvWRKY8 and VvMYB14 (Fig. 3). It has previously been demonstrated that WRKYs are capable of interacting with other proteins including WRKYs and MAP-kinases (Chi *et al.*, 2013; Yang *et al.*, 2017*b*). Although it lacked detailed characterization, a previous Y2H screening analysis has shown that soybean GmWRKY53 interacts with GmMYB114 (Tripathi *et al.*, 2015). Using transient expression assays, Vannozzi *et al.* (2018) showed that VvWRKY3 acts synergistically with VvMYB14 to control the *VvSTS29* promoter, although it has no effect on the promoter by itself. However, the authors excluded any direct interaction between both proteins and further suggested the involvement of an additional ‘bridge’ protein. Here, we demonstrated that the interaction between VvWRKY8 and VvMYB14 was directly mediated through the N-terminal domains of both proteins (Fig. 5).

To further understand how the VvWRKY8–VvMYB14 interaction influences Res biosynthesis, we investigated its effect on the activity of *proVvSTS15/21*. The results indicated that the interaction inhibited the induction effect of VvMYB14 on *proVvSTS15/21* activity (Fig. 4A, B). Moreover, we showed that the inhibition of VvMYB14 by VvWRKY8 was dose-dependent (Fig. 4C). In addition, we determined that the VvMYB14 DNA-binding domain and activation domain are located in the N-terminal and C-terminal halves of the proteins, respectively (Fig. 5C, D). In summary, our results showed that in grapevine, VvWRKY8 can decrease *VvSTS15/21* expression in order to reduce Res biosynthesis by forming a protein complex with VvMYB14.

When grapevines are subjected to abiotic and biotic stresses, Res is quickly synthesized; however, its accumulation is not constant (Douillet-Breuil *et al.*, 1999; Versari *et al.*, 2001; Ferri *et al.*, 2009; Xi *et al.*, 2015; Xu *et al*., 2015*a*, 2015*b*, 2016). Res is a specialized metabolite with strong antioxidant activity that has a protective effect under stresses; however, accumulation of Res to high levels may damage plant development. Therefore, grapevine might have developed a precise regulatory mechanism that relies on a number of activators and repressors in order to balance Res biosynthesis. We found that overexpression of *VvMYB14* resulted in the accumulation of Res and in the up-regulation of *VvWRKY8* expression in grapevine leaves (Fig. 7A, B). However, VvMYB14 did not trans-activate the *VvWRKY8* promoter (Supplementary Fig. S5). In addition, as shown by Supplementary Fig. S1, upon UV-C treatment the expression peak of *VvMYB14* preceded those of *VvSTS*s and *VvWRKY8*. Summing up these results, we speculate that the accumulation of Res results in up-regulation of *VvWRKY8* expression, i.e. there is feedback control between Res and *VvWRKY8*. This hypothesis was confirmed by the 2-fold stimulation of *VvWRKY8* expression when exogenous *trans*-Res was added to grapevine suspension cells (Fig. 7C). Moreover, when exogenous *trans*-Res was sprayed onto tobacco leaves transiently expressing *proVvWRKY8::LUC*, the promoter activity was increased by 3-fold (Fig. 7D).

**Fig. 8.**
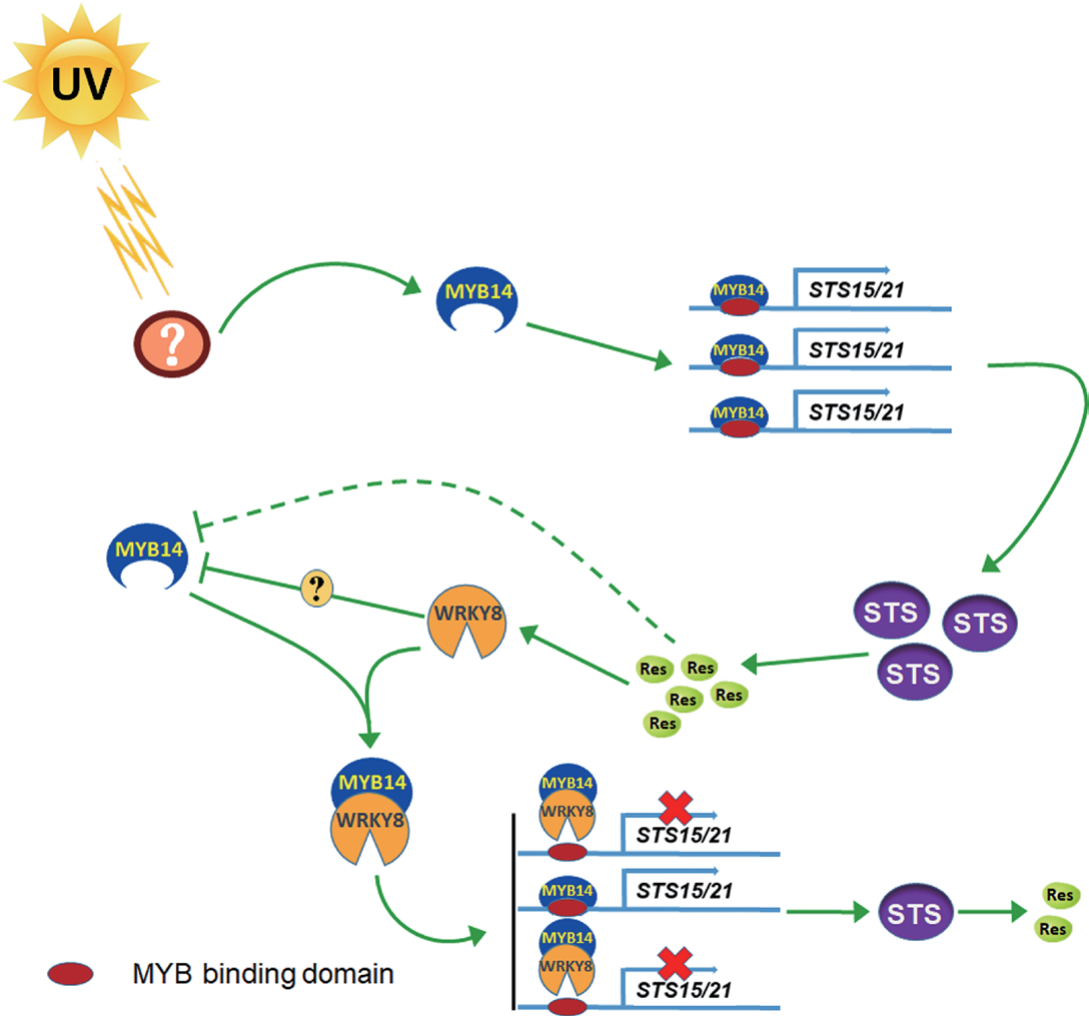
Hypothetical model for the mode of action of the VvMYB14-*VvSTS15/21*-Res-VvWRKY8 regulating loop for the fine-tuning of resveratrol (Res) biosynthesis in grapevine. When plants are exposed to an external signal such as UV-C irradiation, the transcription of *VvMYB14* is activated. VvMYB14 promotes the transcription of *VvSTS15/21*, and Res biosynthesis is stimulated. After the Res concentration reaches a threshold level, it activates the transcription of *VvWRKY8.* Thereafter, the N-termini-mediated VvWRKY8–VvMYB14 interaction inhibits the binding of VvMYB14 to the *VvSTS15/21* promoter, thus resulting in a reduction of Res concentration. In addition, VvWRKY8 can indirectly repress the transcription of *VvMYB14*, and Res itself may also decrease the expression or transcript stability of *VvMYB14*.

Taken together, our results indicate the existence of negative feedback involving the activator VvMYB14, the key enzymes VvSTS15/21, the product Res, and the negative regulator VvWRKY8. Thus, the VvMYB14-VvSTS15/21-Res-VvWRKY8 regulatory loop enables the precise control of *VvSTS15/21* expression levels that are necessary for the regulation of Res biosynthesis (Fig. 8). Data from the literature also show the existence of negative feedback in the control of phytohormone biosynthesis. For example, in peanut, AhAREB1 and AhNAC2 form a protein complex to mediate ABA-dependent negative feedback regulation of *AhNCED1* transcription (Liu *et al.*, 2016). Another mechanism that fine-tunes metabolite biosynthesis is the degradation of transcriptional regulators (Patra *et al.*, 2013). We speculate that degradation of VvWRKY8 allows the de-repression of VvMYB14, and leads to the activation of the Res biosynthetic pathway. Indeed, our Y2H screening using VvWRKY8 as a bait led to the identification of several E3 ubiquitin ligases (Supplementary Fig. S7A, Supplementary Table S4), suggesting that the ubiquitin ligase proteasome pathway may be involved in the control of the VvMYB14-VvSTS15/21-Res-VvWRKY8 regulatory loop. In addition, treatment of tobacco leaves transiently expressing *VvWRKY8* with the proteasome inhibitor MG132 strongly increased the amounts of VvWRKY8, further supporting the notion of VvWRKY8 undergoing ubiquitin proteasome degradation (Supplementary Fig. S7B). However, the relationships among Res and the expression or transcript stability of *VvMYB14* and *VvWRKY8* remain open to question. As shown in Figs 2A and 6C, overexpression of *VvWRKY8* resulted in a decrease in *VvMYB14* expression. However, VvWRKY8 could not directly regulate *VvMYB14* expression (Supplementary Fig. S4). Therefore, it is possible that additional bridge proteins could be involved in the *VvMYB14* expression patterns controlled by VvWRKY8. Furthermore, because exogenous Res decreased *VvMYB14* expression (Supplementary Fig. 7C, we cannot exclude the possibility that Res accumulation itself represses *VvMYB14* expression or transcript stability rather than through VvWRKY8

In summary, this work increases our understanding of the regulatory mechanisms controlling Res biosynthesis and demonstrates that the VvMYB14-VvSTS15/21-Res-VvWRKY8 regulatory loop is an important mechanism that controls Res biosynthesis in grapevine. Our work provides new insights into the complex regulatory network that governs the biosynthesis of a highly valuable specialized metabolite.

## Supplementary data

Supplementary data are available at *JXB* online.

Fig. S1. Effects of UV-C treatment on *VvSTS*s, *VvMYB14*, and *VvWRKY8* expression in grapevine leaves.

Fig. S2. Phylogenetic relationships of VvWRKY8 with other plant WRKYs involved in the regulation of specialized metabolism and stress tolerance.

Fig. S3. Sequence analysis of *proVvSTS15* and *proVvSTS21*.

Fig. S4. VvWRKY8 does not bind to or activate the promoters of *VvSTS15/21* and *VvMYB14*.

Fig. S5. VvMYB14 does not bind to or activate the promoter of *VvWRKY8*.

Fig. S6. Identification of *V. vinifera.* cv. ‘Thompson Seedless’ stable transgenic lines.

Fig. S7. Proteasome degradation of VvWRKY8.

Table S1. List of primers used in this study.

Table S2. Amino acid sequences used for alignment in this study.

Table S3. Effect of addition of exogenous *trans*-Res on Res concentrations in grapevine ‘41B’ suspension cells and tobacco leaves.

Table S4. Interacting proteins of VvWRKY8 as determined by screening a yeast two-hybrid library.

## Supplementary Material

supplementary Figures S1-S7 Tables S1-S4

## References

[CIT0001] Adrian M, Jeandet P 2012 Effects of resveratrol on the ultrastructure of *Botrytis cinerea* conidia and biological significance in plant/pathogen interactions. Fitoterapia 83, 1345–1350. 22516542 10.1016/j.fitote.2012.04.004

[CIT0002] Adrian M, Jeandet P, Veneau J, Weston LA, Bessis R 1997 Biological activity of resveratrol, a stilbenic compound from grapevines, against *Botrytis cinerea*, the causal agent for gray mold. Journal of Chemical Ecology 23, 1689–1702.

[CIT0003] Amato A, Cavallini E, Zenoni S, Finezzo L, Begheldo M, Ruperti B, Tornielli GB 2016 A grapevine TTG2-like WRKY transcription factor is involved in regulating vacuolar transport and flavonoid biosynthesis. Frontiers in Plant Science 7, 1979. 28105033 10.3389/fpls.2016.01979 PMC5214514

[CIT0004] Austin MB, Noel JP 2003 The chalcone synthase superfamily of type III polyketide synthases. Natural Product Reports 20, 79–110. 12636085 10.1039/b100917f

[CIT0005] Cai R, Dai W, Zhang C, Wang Y, Wu M, Zhao Y, Ma Q, Xiang Y, Cheng B 2017 The maize WRKY transcription factor ZmWRKY17 negatively regulates salt stress tolerance in transgenic Arabidopsis plants. Planta 246, 1215–1231. 28861611 10.1007/s00425-017-2766-9

[CIT0006] Chen H, Lai Z, Shi J, Xiao Y, Chen Z, Xu X 2010 Roles of arabidopsis WRKY18, WRKY40 and WRKY60 transcription factors in plant responses to abscisic acid and abiotic stress. BMC Plant Biology 10, 281. 21167067 10.1186/1471-2229-10-281 PMC3023790

[CIT0007] Chen M, Yan T, Shen Q, et al 2017 GLANDULAR TRICHOME-SPECIFIC WRKY 1 promotes artemisinin biosynthesis in *Artemisia annua*. New Phytologist 214, 304–316. 28001315 10.1111/nph.14373

[CIT0008] Chi Y, Yang Y, Zhou Y, Zhou J, Fan B, Yu JQ, Chen Z 2013 Protein–protein interactions in the regulation of WRKY transcription factors. Molecular Plant 6, 287–300. 23455420 10.1093/mp/sst026

[CIT0009] Douillet-Breuil AC, Jeandet P, Adrian M, Bessis R 1999 Changes in the phytoalexin content of various *Vitis* spp. in response to ultraviolet C elicitation. Journal of Agricultural and Food Chemistry 47, 4456–4461. 10552833 10.1021/jf9900478

[CIT0010] Eulgem T, Rushton PJ, Robatzek S, Somssich IE 2000 The WRKY superfamily of plant transcription factors. Trends in Plant Science 5, 199–206. 10785665 10.1016/s1360-1385(00)01600-9

[CIT0011] Fang L, Hou Y, Wang L, Xin H, Wang N, Li S 2014 *Myb14*, a direct activator of *STS*, is associated with resveratrol content variation in berry skin in two grape cultivars. Plant Cell Reports 33, 1629–1640. 24948530 10.1007/s00299-014-1642-3

[CIT0012] Ferri M, Tassoni A, Franceschetti M, Righetti L, Naldrett MJ, Bagni N 2009 Chitosan treatment induces changes of protein expression profile and stilbene distribution in *Vitis vinifera* cell suspensions. Proteomics 9, 610–624. 19132683 10.1002/pmic.200800386

[CIT0013] Grefen C, Blatt MR 2012 A 2in1 cloning system enables ratiometric bimolecular fluorescence complementation (rBiFC). BioTechniques 53, 311–314. 23066669 10.2144/000113941

[CIT0014] Guillaumie S, Mzid R, Méchin V, Léon C, Hichri I, Destrac-Irvine A, Trossat-Magnin C, Delrot S, Lauvergeat V 2010 The grapevine transcription factor WRKY2 influences the lignin pathway and xylem development in tobacco. Plant Molecular Biology 72, 215–234. 19902151 10.1007/s11103-009-9563-1

[CIT0015] Gutha LR, Casassa LF, Harbertson JF, Naidu RA 2010 Modulation of flavonoid biosynthetic pathway genes and anthocyanins due to virus infection in grapevine (*Vitis vinifera* L.) leaves. BMC Plant Biology 10, 187. 20731850 10.1186/1471-2229-10-187 PMC2956537

[CIT0016] Han Y, Dang R, Li J, Jiang J, Zhang N, Jia M, Wei L, Li Z, Li B, Jia W 2015 FaSnRK2.6, an ortholog of open stomata 1, is a negative regulator of strawberry fruit development and ripening. Plant Physiology 167, 915–930. 25609556 10.1104/pp.114.251314 PMC4348756

[CIT0017] Hecker A, Wallmeroth N, Peter S, Blatt MR, Harter K, Grefen C 2015 Binary 2in1 vectors improve *in planta* (co)localization and dynamic protein interaction studies. Plant Physiology 168, 776–787. 25971551 10.1104/pp.15.00533 PMC4741326

[CIT0018] Hellens RP, Allan AC, Friel EN, Bolitho K, Grafton K, Templeton MD, Karunairetnam S, Gleave AP, Laing WA 2005 Transient expression vectors for functional genomics, quantification of promoter activity and RNA silencing in plants. Plant Methods 1, 13. 16359558 10.1186/1746-4811-1-13 PMC1334188

[CIT0019] Höll J, Vannozzi A, Czemmel S, D’Onofrio C, Walker AR, Rausch T, Lucchin M, Boss PK, Dry IB, Bogs J 2013 The R2R3-MYB transcription factors MYB14 and MYB15 regulate stilbene biosynthesis in *Vitis vinifera*. The Plant Cell 25, 4135–4149. 24151295 10.1105/tpc.113.117127 PMC3877794

[CIT0020] Ishiguro S, Nakamura K 1994 Characterization of a cDNA encoding a novel DNA-binding protein, SPF1, that recognizes SP8 sequences in the 5′ upstream regions of genes coding for sporamin and beta-amylase from sweet potato. Molecular & General Genetics 244, 563–571. 7969025 10.1007/BF00282746

[CIT0021] Kalantari H, Das DK 2010 Physiological effects of resveratrol. BioFactors 36, 401–406. 20623511 10.1002/biof.100

[CIT0022] Karimi M, Inzé D, Depicker A 2002 GATEWAY vectors for *Agrobacterium*-mediated plant transformation. Trends in Plant Science 7, 193–195. 11992820 10.1016/s1360-1385(02)02251-3

[CIT0023] Kato N, Dubouzet E, Kokabu Y, Yoshida S, Taniguchi Y, Dubouzet JG, Yazaki K, Sato F 2007 Identification of a WRKY protein as a transcriptional regulator of benzylisoquinoline alkaloid biosynthesis in *Coptis japonica*. Plant & Cell Physiology 48, 8–18. 17132631 10.1093/pcp/pcl041

[CIT0024] Langcake P, Pryce RJ 1976 Production of resveratrol by *Vitis vinifera* and other members of Vitaceae as a response to infection or injury. Physiological Plant Pathology 9, 77–86.

[CIT0025] Li B, Zhao Y, Liang L, et al 2012 Purification and characterization of ZmRIP1, a novel reductant-inhibited protein tyrosine phosphatase from maize. Plant Physiology 159, 671–681. 22529284 10.1104/pp.111.191510 PMC3375933

[CIT0026] Li P, Song A, Gao C, Wang L, Wang Y, Sun J, Jiang J, Chen F, Chen S 2015 Chrysanthemum WRKY gene *CmWRKY17* negatively regulates salt stress tolerance in transgenic chrysanthemum and Arabidopsis plants. Plant Cell Reports 34, 1365–1378. 25893877 10.1007/s00299-015-1793-x

[CIT0027] Liu S, Li M, Su L, Ge K, Li L, Li X, Liu X, Li L 2016 Negative feedback regulation of ABA biosynthesis in peanut (*Arachis hypogaea*): a transcription factor complex inhibits *AhNCED1* expression during water stress. Scientific Reports 6, 37943. 27892506 10.1038/srep37943 PMC5124963

[CIT0028] Naoumkina MA, He X, Dixon RA 2008 Elicitor-induced transcription factors for metabolic reprogramming of secondary metabolism in *Medicago truncatula*. BMC Plant Biology 8, 132. 19102779 10.1186/1471-2229-8-132 PMC2628384

[CIT0029] Nonomura S, Kanagawa H, Makimoto A 1963 Chemical constituents of polygonaceous plants. I. Studies on the components of ko-j o-kon. (*Polygonum cuspidatum* Sieb. et Zucc.). [In Japanese.] Yakugaku Zasshi (Journal of the Pharmaceutical Society of Japan) 83, 988–990. 14089847

[CIT0030] Pangeni R, Sahni JK, Ali J, Sharma S, Baboota S 2014 Resveratrol: review on therapeutic potential and recent advances in drug delivery. Expert Opinion on Drug Delivery 11, 1285–1298. 24830814 10.1517/17425247.2014.919253

[CIT0031] Patra B, Pattanaik S, Yuan L 2013 Ubiquitin protein ligase 3 mediates the proteasomal degradation of GLABROUS 3 and ENHANCER OF GLABROUS 3, regulators of trichome development and flavonoid biosynthesis in Arabidopsis. The Plant Journal 74, 435–447. 23373825 10.1111/tpj.12132

[CIT0032] Qin Z, Lv H, Zhu X, Meng C, Quan T, Wang M, Xia G 2013 Ectopic expression of a wheat WRKY transcription factor gene *TaWRKY71-1* results in hyponastic leaves in *Arabidopsis thaliana*. PLoS ONE 8, e63033. 23671653 10.1371/journal.pone.0063033 PMC3650005

[CIT0033] Qiu D, Xiao J, Xie W, Liu H, Li X, Xiong L, Wang S 2008 Rice gene network inferred from expression profiling of plants overexpressing OsWRKY13, a positive regulator of disease resistance. Molecular Plant 1, 538–551. 19825559 10.1093/mp/ssn012

[CIT0034] Schefe JH, Lehmann KE, Buschmann IR, Unger T, Funke-Kaiser H 2006 Quantitative real-time RT-PCR data analysis: current concepts and the novel “gene expression’s *C*_T_ difference” formula. Journal of Molecular Medicine 84, 901–910. 16972087 10.1007/s00109-006-0097-6

[CIT0035] Schluttenhofer C, Yuan L 2015 Regulation of specialized metabolism by WRKY transcription factors. Plant Physiology 167, 295–306. 25501946 10.1104/pp.114.251769 PMC4326757

[CIT0036] Sheen J, Hwang S, Niwa Y, Kobayashi H, Galbraith DW 1995 Green-fluorescent protein as a new vital marker in plant cells. The Plant Journal 8, 777–784. 8528289 10.1046/j.1365-313x.1995.08050777.x

[CIT0037] Singh AK, Kumar SR, Dwivedi V, Rai A, Pal S, Shasany AK, Nagegowda DA 2017 A WRKY transcription factor from *Withania somnifera* regulates triterpenoid withanolide accumulation and biotic stress tolerance through modulation of phytosterol and defense pathways. New Phytologist 215, 1115–1131. 28649699 10.1111/nph.14663

[CIT0038] Takaoka M 1939 Resveratrol, a new phenolic compound, from *Veratrum grandiflorum*. Journal of the Chemical Society of Japan 60, 1090–1100.

[CIT0039] **The French–Italian Public Consortium for Grapevine Genome Characterization** 2007 The grapevine genome sequence suggests ancestral hexaploidization in major angiosperm phyla. Nature 449, 463–467. 17721507 10.1038/nature06148

[CIT0040] Thompson JD, Gibson TJ, Plewniak F, Jeanmougin F, Higgins DG 1997 The CLUSTAL_X windows interface: flexible strategies for multiple sequence alignment aided by quality analysis tools. Nucleic Acids Research 25, 4876–4882. 9396791 10.1093/nar/25.24.4876 PMC147148

[CIT0041] Tripathi P, Rabara RC, Choudhary MK, Miller MA, Huang YS, Shen QJ, Blachon S, Rushton PJ 2015 The interactome of soybean GmWRKY53 using yeast 2-hybrid library screening to saturation. Plant Signaling & Behavior 10, e1028705. 26102586 10.1080/15592324.2015.1028705 PMC4623026

[CIT0042] van Verk MC, Pappaioannou D, Neeleman L, Bol JF, Linthorst HJ 2008 A novel WRKY transcription factor is required for induction of *PR-1a* gene expression by salicylic acid and bacterial elicitors. Plant Physiology 146, 1983–1995. 18263781 10.1104/pp.107.112789 PMC2287365

[CIT0043] Vannozzi A, Dry IB, Fasoli M, Zenoni S, Lucchin M 2012 Genome-wide analysis of the grapevine stilbene synthase multigenic family: genomic organization and expression profiles upon biotic and abiotic stresses. BMC Plant Biology 12, 130. 22863370 10.1186/1471-2229-12-130 PMC3433347

[CIT0044] Vannozzi A, Wong DCJ, Höll J, Hmmam I, Matus JT, Bogs J, Ziegler T, Dry I, Barcaccia G, Lucchin M 2018 Combinatorial regulation of stilbene synthase genes by WRKY and MYB transcription factors in grapevine (*Vitis vinifera* L.). Plant & Cell Physiology 59, 1043–1059. 29529275 10.1093/pcp/pcy045

[CIT0045] Versari A, Parpinello GP, Tornielli GB, Ferrarini R, Giulivo C 2001 Stilbene compounds and stilbene synthase expression during ripening, wilting, and UV treatment in grape cv. Corvina. Journal of Agricultural and Food Chemistry 49, 5531–5536. 11714355 10.1021/jf010672o

[CIT0046] Wang H, Avci U, Nakashima J, Hahn MG, Chen F, Dixon RA 2010 Mutation of WRKY transcription factors initiates pith secondary wall formation and increases stem biomass in dicotyledonous plants. Proceedings of the National Academy of Sciences, USA 107, 22338–22343. 10.1073/pnas.1016436107 PMC3009815 21135241

[CIT0047] Wang L, Zhu W, Fang L, Sun X, Su L, Liang Z, Wang N, Londo JP, Li S, Xin H 2014 Genome-wide identification of WRKY family genes and their response to cold stress in *Vitis vinifera*. BMC Plant Biology 14, 103. 24755338 10.1186/1471-2229-14-103 PMC4021059

[CIT0048] Weiskirchen S, Weiskirchen R 2016 Resveratrol: how much wine do you have to drink to stay healthy?Advances in Nutrition 7, 706–718. 27422505 10.3945/an.115.011627 PMC4942868

[CIT0049] Xi H, Ma L, Liu G, Wang N, Wang J, Wang L, Dai Z, Li S, Wang L 2014 Transcriptomic analysis of grape (*Vitis vinifera* L.) leaves after exposure to ultraviolet C irradiation. PLoS ONE 9, e113772. 25464056 10.1371/journal.pone.0113772 PMC4252036

[CIT0050] Xi HF, Ma L, Wang LN, Li SH, Wang LJ 2015 Differential response of the biosynthesis of resveratrols and flavonoids to UV-C irradiation in grape leaves. New Zealand Journal of Crop and Horticultural Science 43, 163–172.

[CIT0051] Xie Z, Zhang ZL, Zou X, Yang G, Komatsu S, Shen QJ 2006 Interactions of two abscisic-acid induced *WRKY* genes in repressing gibberellin signaling in aleurone cells. The Plant Journal 46, 231–242. 16623886 10.1111/j.1365-313X.2006.02694.x

[CIT0052] Xu A, Zhan JC, Huang WD 2015a Effects of ultraviolet C, methyl jasmonate and salicylic acid, alone or in combination, on stilbene biosynthesis in cell suspension cultures of *Vitis vinifera* L. cv. Cabernet Sauvignon. Plant Cell Tissue and Organ Culture 122, 197–211.

[CIT0053] Xu A, Zhan JC, Huang WD 2015b Oligochitosan and sodium alginate enhance stilbene production and induce defense responses in *Vitis vinifera* cell suspension cultures. Acta Physiologiae Plantarum 37, 144.

[CIT0054] Xu A, Zhan JC, Huang WD 2016 Combined elicitation of chitosan and ultraviolet C enhanced stilbene production and expression of chitinase and beta-1,3-glucanase in *Vitis vinifera* cell suspension cultures. Plant Cell Tissue and Organ Culture 124, 105–117.

[CIT0055] Xu W, Yu Y, Ding J, Hua Z, Wang Y 2010 Characterization of a novel stilbene synthase promoter involved in pathogen- and stress-inducible expression from Chinese wild *Vitis pseudoreticulata*. Planta 231, 475–487. 19937257 10.1007/s00425-009-1062-8

[CIT0056] Xu YH, Wang JW, Wang S, Wang JY, Chen XY 2004 Characterization of GaWRKY1, a cotton transcription factor that regulates the sesquiterpene synthase gene (+)-*δ*-cadinene synthase-A. Plant Physiology 135, 507–515. 15133151 10.1104/pp.104.038612 PMC429402

[CIT0057] Yang CY, Huang YC, Ou SL 2017a ERF73/HRE1 is involved in H_2_O_2_ production via hypoxia-inducible *Rboh* gene expression in hypoxia signaling. Protoplasma 254, 1705–1714. 27995331 10.1007/s00709-016-1064-x

[CIT0058] Yang G, Zhang W, Liu Z, Yi-Maer AY, Zhai M, Xu Z 2017b Both JrWRKY2 and JrWRKY7 of *Juglans regia* mediate responses to abiotic stresses and abscisic acid through formation of homodimers and interaction. Plant Biology 19, 268–278. 27860167 10.1111/plb.12524

[CIT0059] Yokotani N, Sato Y, Tanabe S, et al 2013 WRKY76 is a rice transcriptional repressor playing opposite roles in blast disease resistance and cold stress tolerance. Journal of Experimental Botany 64, 5085–5097. 24043853 10.1093/jxb/ert298 PMC3830488

[CIT0060] Yu O, Jez JM 2008 Nature’s assembly line: biosynthesis of simple phenylpropanoids and polyketides. The Plant Journal 54, 750–762. 18476876 10.1111/j.1365-313X.2008.03436.x

[CIT0061] Zhou Q, Dai L, Cheng S, He J, Wang D, Zhang J, Wang Y 2014 A circulatory system useful both for long-term somatic embryogenesis and genetic transformation in *Vitis vinifera* L. cv. Thompson seedless. Plant Cell Tissue and Organ Culture 118, 157–168.

