# Supplementary material for: VvWRKY8 represses stilbene synthase genes through direct interaction with VvMYB14 to control resveratrol biosynthesis in grapevine
Source: J Exp Bot. 2018 Nov 16;70(2):715–29. doi: 10.1093/jxb/ery401 (PMC6322584; doi:10.1093/jxb/ery401)
Supplement: supplementary Figures S1-S7 Tables S1-S4 [file ery401_suppl_supplementary_figures_s1-s7_tables_s1-s4.pdf]

Article title: VvWRKY8 negatively regulates VvSTS through direct interaction with VvMYB14 to balance resveratrol biosynthesis in grapevine

Authors: Jinzhu Jiang, Huifen Xi, Zhanwu Dai, Fatma Lecourieux, Ling Yuan, Xianju Liu, Barunava Patra, Yongzan Wei, Shaohua Li and Lijun Wang

The following Supporting Information is available for this article:

Fig. S1 Effect of UV-C treatment on *VvSTSs, VvMYB14* and *VvWRKY8* expression in grapevine leaves.

Fig.S2 Phylogenetic relationship of VvWRKY8 with other plant WRKYs involved in the regulation of specialized metabolism and stress tolerance.

Fig.S3 Sequence analysis of *proVvSTS15* and *proVvSTS21*.

Fig. S4 VvWRKY8 does not bind to nor activate the promoters of *VvSTS15/21* and *VvMYB14*.

Fig. S5 VvMYB14 does not bind to nor activate the promoter of *VvWRKY8*.

Fig. S6 Identification of *V. vinifera.* cv. ‘Thompson Seedless’ stable transgenic lines.

Fig. S7 Proteasome degradation of VvWRKY8.

Table S1 The primers used in this article.

Table S2 The amino acid sequences used for alignment in this article.

Table S3 Effect of exogenous *trans*-Res supplement or spraying on Res concentrations in ‘41B’ grapevine suspension cells or tobacco leaves.

Table S4 The interacting proteins of VvWRKY8 by screening yeast two-hybrid library.

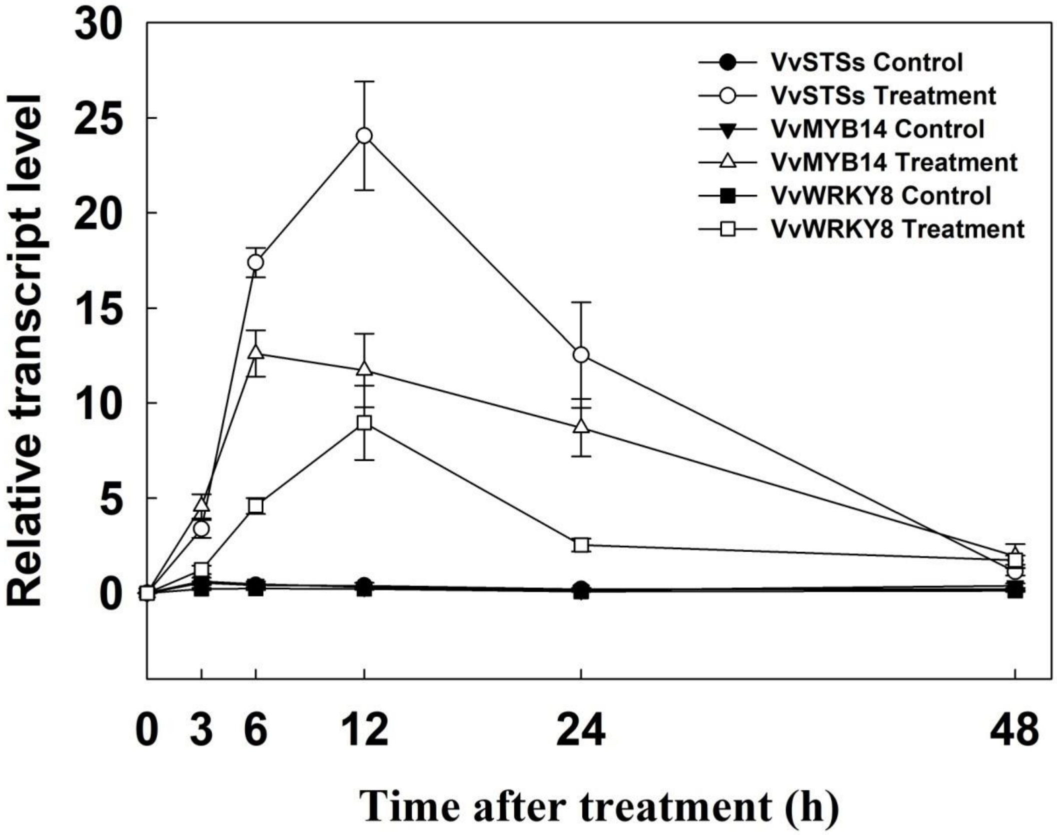

Fig. S1 Effect of UV-C treatment on *VvSTSs, VvMYB14* and *VvWRKY8* expression in grapevine leaves. The primers used for detecting *VvSTSs* expression levels were designed in a region conserved in the 25 *VvSTS* mRNA sequences. Expression levels of genes were normalized to *VvActin7*.

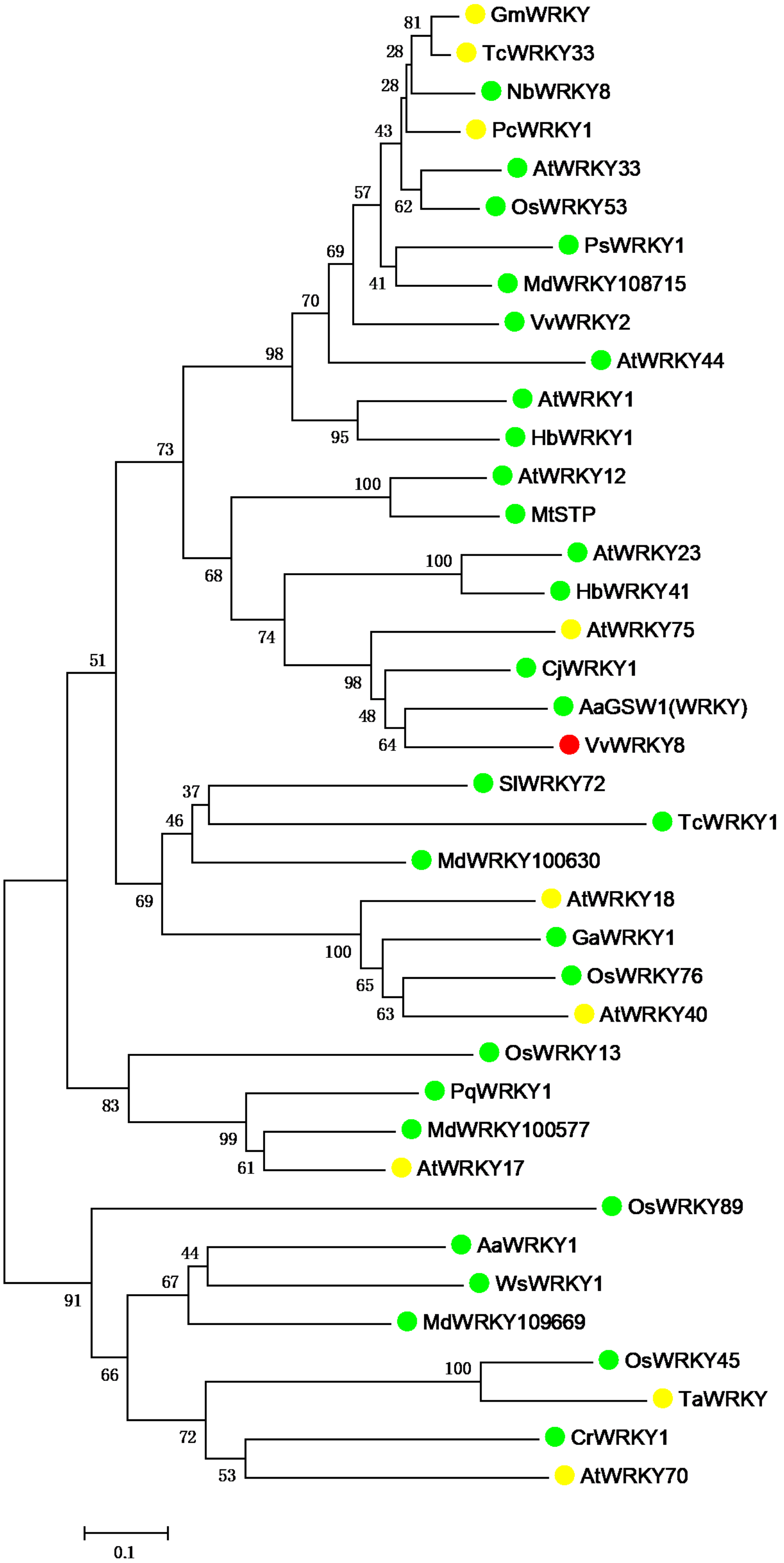
GmWRKY
TcWRKY33
NbWRKY8
PcWRKY1
AtWRKY33
OsWRKY53
PsWRKY1
MdWRKY108715
VvWRKY2
AtWRKY44
AtWRKY1
HbWRKY1
AtWRKY12
MtSTP
AtWRKY23
HbWRKY41
AtWRKY75
CjWRKY1
AaGSW1(WRKY)
VvWRKY8
SlWRKY72
TcWRKY1
MdWRKY100630
AtWRKY18
GaWRKY1
OsWRKY76
AtWRKY40
OsWRKY13
PqWRKY1
MdWRKY100577
AtWRKY17
OsWRKY89
AaWRKY1
WsWRKY1
MdWRKY109669
OsWRKY45
TaWRKY
CrWRKY1
AtWRKY70
0.1

Fig. S2 Phylogenetic relationship of VvWRKY8 with other plant WRKYs involved in the regulation of specialized metabolism and stress tolerance. The tree was constructed using MEGA7 and the statistical reliability of individual nodes of the tree was assessed by bootstrap analyses with 1000 replicates. Names of sequences with green and yellow circles represent WRKYs involved in specialized metabolisms and biotic/abiotic stresses, respectively. VvWRKY8 characterized in this study is shown with a red circle. The GenBank accession numbers of other plant WRKYs were obtained from Singh *et al*. (2017).

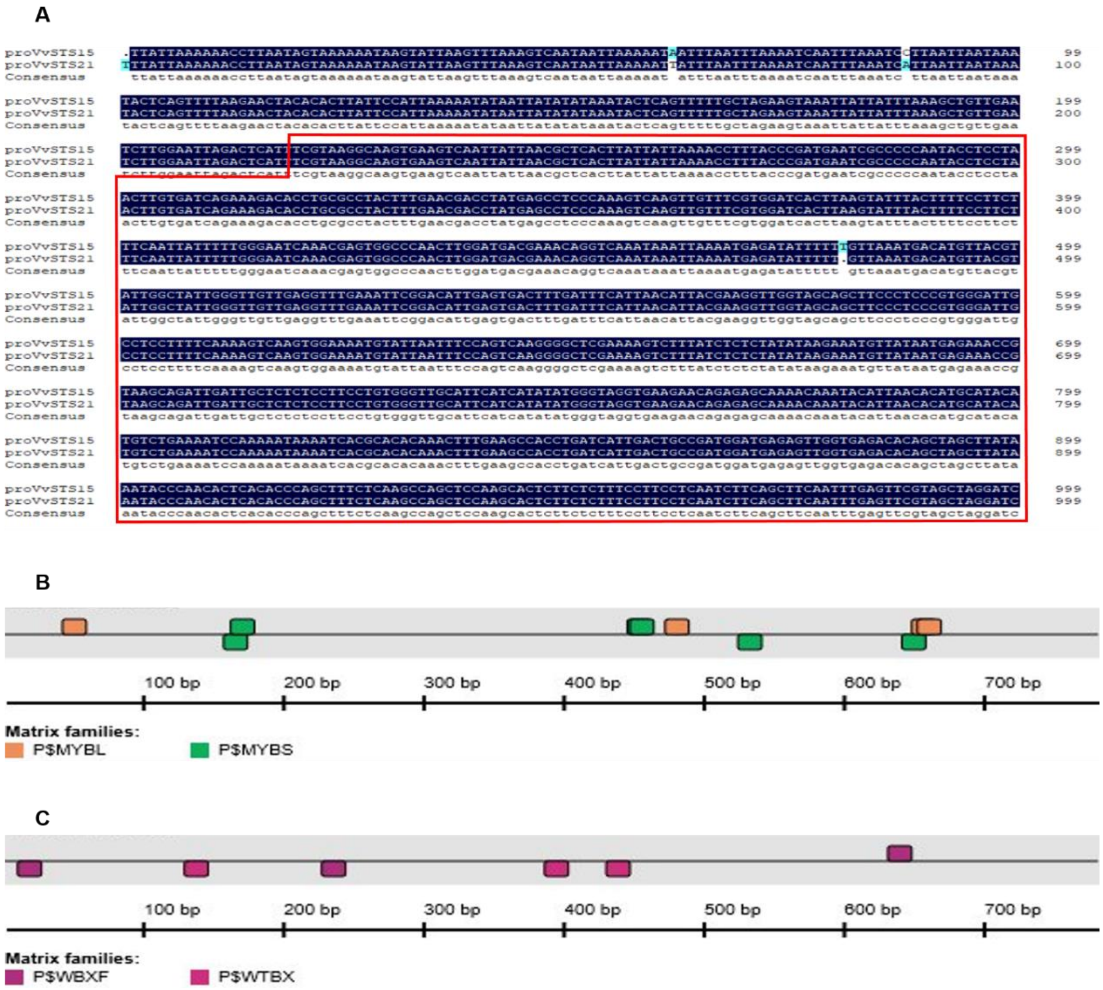

Fig. S3 Sequence analysis of *proVvSTS15* and *proVvSTS21*. A, Similarities between the 1000 bp promoter sequences of *VvSTS15* and *VvSTS21*. The 781 bp similar sequence (in red box) was cloned for further experiment (named as *proVvSTS15/21*). **B, C,** Diagram showing WRKY and MYB binding motif in *proVvSTS15/21*. The *cis*-regulatory element screening of *proVvSTS15/21* was done using MatInspector software (http://www. genomatix. de/solutions/genomatix-software-suite.html).

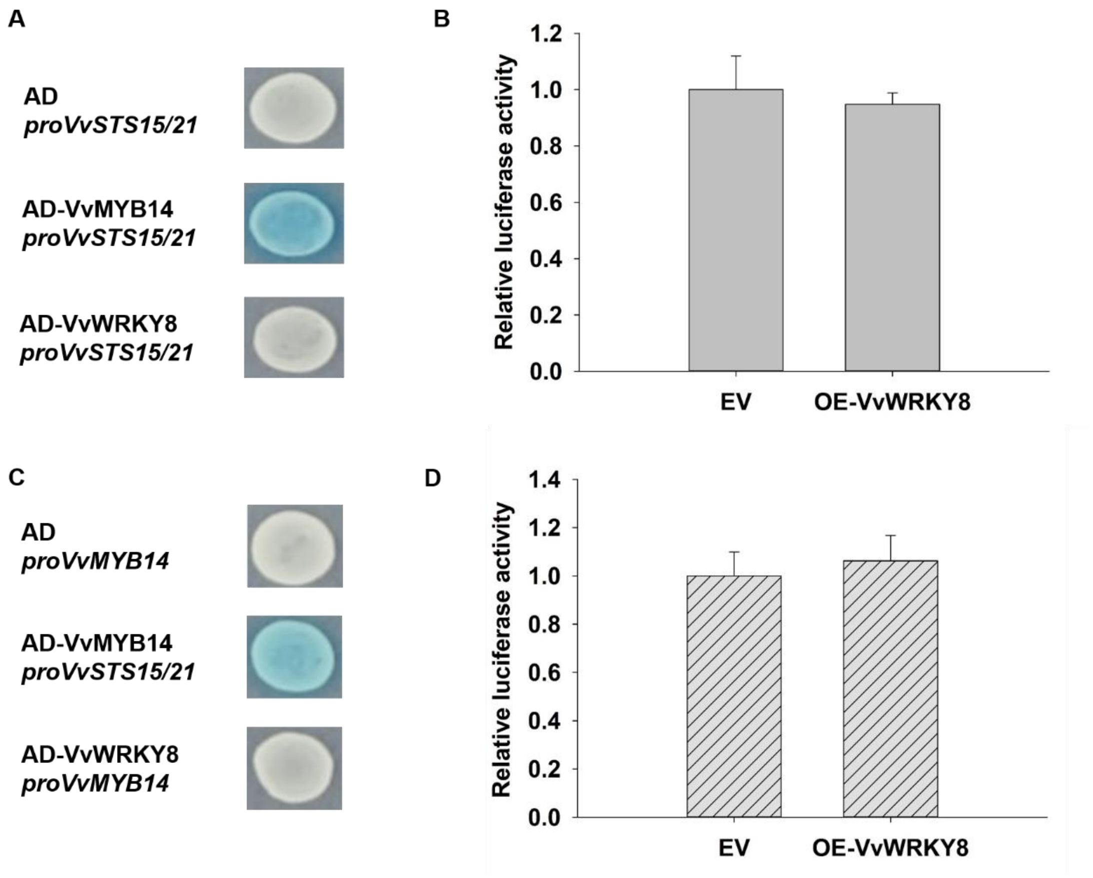

Fig. S4 VvWRKY8 does not bind to nor activate the promoters of *VvSTS15/21* and *VvMYB14*. A, C, Yeast one-hybrid assay. *VvWRKY8* was fused with the B42 activation domain (AD), co-transformed with the *proVvSTS15/21:LacZ* or *proVvMYB14:LacZ* reporters into yeast cells. The combination of VvMYB14 protein and *proVvSTS15/21:LacZ* works as the positive control. The transformants were further grown on SD/-Trp/-Ura selection media supplied with 20 g L^{-1} X-Gal for color development. A, *proVvSTS15/21:LacZ*; C, *proVvMYB14:LacZ*. **B, D,** Transient expression assays of *proVvSTS15/21:LUC* and *proVvMYB14:LUC* reporter with VvWRKY8 transcriptional effector in tobacco leaves. Activities of LUC protein were normalized to renilla LUC and were represented as activities relative to EV that was set to 1. B, *proVvSTS15/21:LUC*; D, The *proVvMYB14:LUC*. Bars represent standard error (SE). Data from three independent replicates are shown as mean ± SE (Student's t-test: *, $P < 0.05$; **, $P < 0.01$).

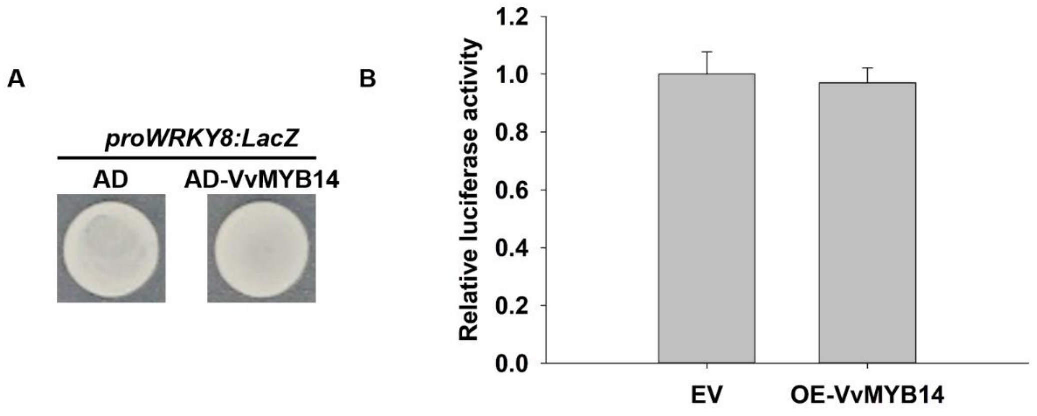

Fig. S5 VvMYB14 does not bind to and activate the promoter of VvWRKY8. A, Yeast one-hybrid assay. The *VvMYB14* was fused to the B42 activation domain (AD), co-transformed with the *proVvWRKY8:LacZ* reporter into yeast cells. The transformants were further grown on SD/-Trp/-Ura selection media supplied with 20 g L^{-1} X-Gal for color development. **B,** Transient expression assay of *proVvWRKY8:LUC* reporter with VvMYB14 transcriptional effector in tobacco leaves. Activities of LUC protein were normalized to renilla LUC and were represented as activities relative to EV that was set to 1. Bars represent standard error (SE). Data from three independent replicates are shown as mean ± SE (Student's t-test: *, $P < 0.05$; **, $P < 0.01$).

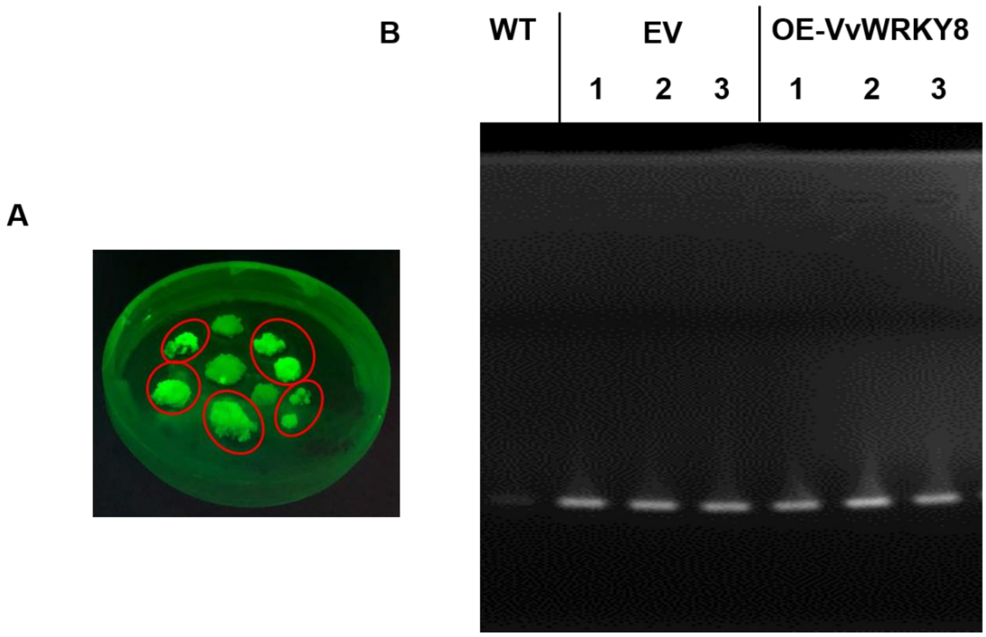

Fig. S6 Identification of *V. vinifera*. cv. ‘Thompson Seedless’ stable transgenic lines. **A,** Using the xenon lamp with 488 nm filter, callus showing GFP fluorescence were subcultured and Res concentration was measured. **B,** RT-PCR detection of GFP expression in stable transgenic grapevine lines. Primers were designed using *GFP* sequence. WT: the wild type *V. vinifera*. cv. ‘Thompson Seedless’ (negative control); EV: empty vector transgenic grapevines expressing GFP alone; OE-VvWRKY8: transgenic grapevines overexpressing VvWRKY8.

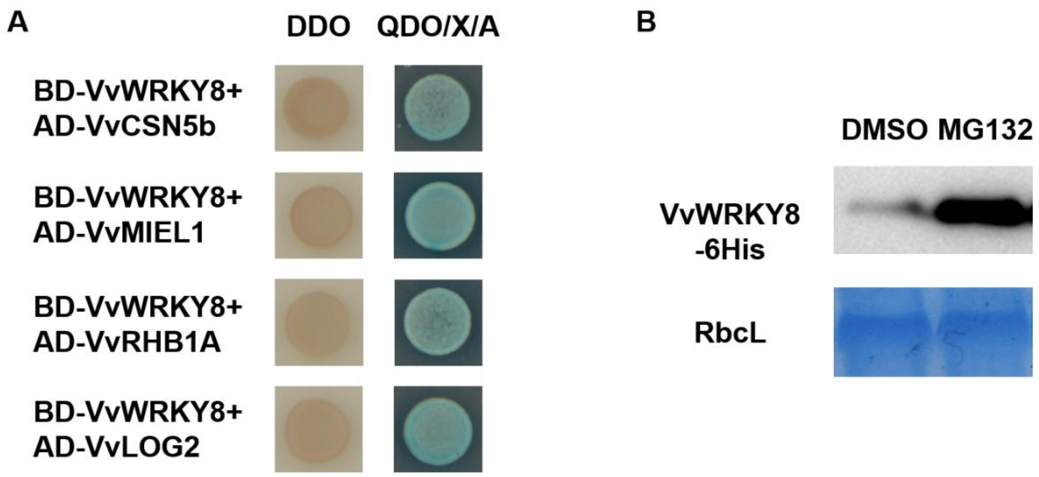

Fig. S7 Proteasome degradation of VvWRKY8. A, VvWRKY8 interacts with candidate proteins identified using yeast two-hybrid screening. These putative interactors are involved in ubiquitin-proteasome degradation system. All transformants were conducted on SD/-Leu/-Trp (DDO) or SD/-Leu/-Trp/-His/-Ade/X-α-Gal (QDO/X) selection media. Interactions were determined on the basis of cell growth and cell color. **B,** Immunoblot analysis of VvWRKY8-6×His protein (by anti-His-tag antibody) transiently expressed in tobacco leaves treated or not with MG132. RbcL, ribulose-1,5-bis-phosphate carboxylase/oxygenase large subunit (loading control). Two days after the tobacco leaves were transiently transformed by VvWRKY8-6×His vector, they were injected with 50 μm MG132 or DMSO. Total protein was extracted after 24 h incubation for immunoblot analyzing the VvWRKY8-6×His proteins using anti-His-tag antibody.

Table S1 The primers used in this article.

Purpose	Primer name	Sequences (5'To3')
Gene isolation	WRKY8-U	ATGGATAGCTTCTCCACTCTCTTTCC
	WKKY8-L	TTAAAAGGAAGCATAAACTTGCATCTG
Subcellular localization	NL-WRKY8-HindIII-U	AAGCTTATGGATAGCTTCTCCACTCTCTTTCCGTGC
	NL-WKKY8-BmaHI-L	GGATCCCGAAAGGAAGCATAAACTTGCATCTG
qRT-PCR analysis	VvSTSs-F	TTAGAAACGCTCAACGTGCCAAGGG
	VvSTSs-R	AATCAGCATAATCAGACTGGTAGAC
	VvSTS15/21-F	GTTGGATCAGATCCAGATGTCTCC
	VvSTS15/21-R	GTGGGTCAAAAGCCTGATTCAAG
	VvWRKY8-F	TCCAAACCCACAAGCCCAGTGA
	VvWRKY8-R	AGTGATGTCTGAGGCGGGAACT
	VvMYB14-F	TCTGAGGCCGGATATCAAAC
	VvMYB14-R	GGGACGCATCAAGAGAGTGT
	VvSPS1-F	TCGTTGAGCAGGTCATTACTGG
	VvSPS1-R	CTGCTTCTTCTGTCGAGCCA
	VvACTIN7-F	CTTGCATCCCTCAGCACCTT
	VvACTIN7-R	TCCTGTGGACAATGGATGGA
Yeast one-hybrid assay	pGAD424- VvWRKY8-F	GATTATGCCTCTCCCGAATTCATGGATAGCTTCTCCACTCTCTTTCC
	pGAD424- VvWRKY8-R	AGAAGTCCAAAGCTTCTCGAGTTAAAAGGAAGCATAAACTTGCATCT
	pGAD424-VvMYB14-F	GATTATGCCTCTCCCGAATTCATGGGGAGAGCTCCTTGTTGT
	pGAD424- VvMYB14-F	AGAAGTCCAAAGCTTCTCGAGTCATATTTCTGATAATTCATGCAACTCC
	pLACZi-kpni-proWRKY8-F	CTTGAATTCGAGCTCGGTACCGAGTGACGACTTTGAGGAGG
	pLACZi-Xhoi-proWRKY8-R	ATACAGAGCACATGCCTCGAGGTTGTTAGAGATGCTTCCAAGG
	pLACZi-kpni-proMYB14-F	GGTACCGTCCAGATGGGGTTGACG
	pLACZi-Xhoi-proMYB14-R	CTCGAGTTTTTCTTTTCTACGTAAGGATTTGAGACT
	pLACZi-kpni-proSTS15/21-F	GGGGTACCTCGTAAGGCAAGTGAAGTCAATTATTAACG
	pLACZi-Xhoi-proSTS15/21-R	CGGATCCGTGATCCTAGCTACGAACTCAAATTG

Yeast two-hybrid assay	WRKY8-NdeI-U	CATATGATGGATAGCTTCTCCACTCTCTTTCC
	WRKY8-1-96-EcoRI-L	GAATTCCTTTCTAATCTTCTTCTCTCCCTTCTTCTTG
	WRKY8-97-189-NdeI-U	CATATGCCCCGATATGCTTTTCAAACCAGGAGC
	WRKY8-EcoRI-L	GAATTCTTAAAAGGAAGCATAAACTTGCATCTGGC
	MYB14-NdeI-U	CATATGGGGAGAGCTCCTTG
	MYB14-1-137-EcoRI-L	GAATTCTTTTTCGACTTGGGACGCATC
	MYB14-138-272-NdeI-U	CATATGCAACAAAACCCCATTAATTCTGCAAC
	MYB14-EcoRI-L	GAATTCTCATATTTCTGATAATTCATGCAACTC
	AD-MIEL1-F	GTACCAGATTACGCTCATATGGGATCCATGGAGTTCCTTCGTCTCAACCT
	AD-MIEL1-23-R	ACGATTCATCTGCAGCTCGAGACTATCAGCCACAGGGGATACA
	AD-VvRHB1A-F	GTACCAGATTACGCTCATATGGGATCCATGGGGGGTTGCTGTTGTTC
	AD-VvRHB1A-R	ACGATTCATCTGCAGCTCGAGCATAATCATGGTATCTTCAAGTATCATT
	AD-VvLOG2-F	GTACCAGATTACGCTCATATGGGATCCATGGGGAATATCGGAAGTAGCA
	AD-VvLOG2-R	ACGATTCATCTGCAGCTCGAGGTCGTCAGACCCATTGCT
	AD-VvCSN5b-F	GTACCAGATTACGCTCATATGGGATCCATGGAACCCTACTCCTTCACATCC
	AD-VvCSN5b-R	ACGATTCATCTGCAGCTCGAGAGTTTCAATCATGGGTTCTGG
Transformation	attB1-VvWRKY8-F	GGGGACAAGTTTGTACAAAAAAGCAGGCTTAATGGATAGCTTCTCCACTC
	attB2- VvWRKY8-R	GGGGACCACTTTGTACAAGAAAGCTGGGTGTTAAAAGGAAGCATAAACTTGCATC
	attB4- VvWRKY8-R	GGGGACAACTTTGTATAGAAAAGTTGGGTGTTAAAAGGAAGCATAAACTTGCATC
	attB3- VvMYB14-F	GGGGACAACTTTGTATAATAAAGTTGTAACCATGGGGAGAGCTCCTTGTTG
	attB1- VvMYB14-F	GGGGACAAGTTTGTACAAAAAAGCAGGCTTAATGGGGAGAGCTCCATGTTGT
	attB2- VvMYB14-R	GGGGACCACTTTGTACAAGAAAGCTGGGTGTCATATTTCTGATAATTCATGCAACTCCC
Transient luciferase expression assay	pGREEN-luc-proWRKY8-F	GTCGACGGTATCGATAAGCTTGAGTGACGACTTTGAGGAGG
	pGREEN-luc-proWRKY8-R	TGTTTTTGGCGTCTTCCATGGGTTGTTAGAGATGCTTCCAAGG
	pGREEN-luc-proMYB14-F	CTATAGGGCGAATTGGGTACCGTCCAGATGGGGTTGACGG
	pGREEN-luc-proMYB14-R	TGTTTTTGGCGTCTTCCATGGCCCCATTTTTTCTTTTCTACGTAAG
	pGREEN-luc-proSTS15/21-F	GGGGTACCTCGTAAGGCAAGTGAAGTCAATTATTAACG
	pGREEN-luc-proSTS15/21-R	CGGATCCGTGATCCTAGCTACGAACTCAAATTG

Table S2 The amino acid sequences used for alignment in this article.

Gene name	Amino acid sequences
AaGSW1	MDNQLDAMFHYSSSPTSPPPQADVSSYLSLNMANQYNNSHADKKHYS<br>NYEQSDVSRSTSSFGTGESSELNMSIMGSGKVNVKKGEKKIRKPKYAFQ<br>TRSQVDILDDGYRWRKYGQKAVKNNKFPRSYYRCTQQGCNVKKQVQR<br>LSKDEGVVVTTYEGMHTHPIERSTDNFEHILTQMQIYSSS
CjWRKY1	MDNYPILFSSPSSSSVAATTSHFPSYMVNNNHVFSDVHSNNQNGLFVAE<br>MKSEIDVIPPSSSNNSNSSGSNGGGDNTDMKSDKKKVDKKVRNPRYAFQ<br>TRSQVDILDDGYRWRKYGQKAVKNNKHPRSYYRCTHQGCNVKKQVQ<br>RLSKDEGVVVTTYEGVHAHPIEKSTDNFENILSQMQIYTGY
AtWRKY75	MEGYDNGSLYAPFLSLKSHSKPELHQGEEESSKVRSEGCSKSVESSKKK<br>GKKQRYAFQTRSQVDILDDGYRWRKYGQKAVKNNKFPRSYYRCTYGG<br>CNVKKQVQRLTVDQEVVVTTYEGVHSHPIEKSTENFEHILTQMQIYSSF
VvWRKY8	MDSFSTLFPCPPSTSSPSPFSFLSMVNNSSHHDFQTHKPSDFLGLMSGTTA<br>TAASMEVPASDITNVDSLQAKGLLGSDDGEVKSCGKKKGEKKIRKPRYA<br>FQTRSQVDILDDGYRWRKYGQKAVKNNRFPRSYYRCTHQGCNVKKQV<br>QRLSKDEGIVVTTYEGMHSHQIEKSTDNFEHILSQMQVYASF

Table S3 Effect of exogenous *trans*-Res supplement or spraying on Res concentrations in '41B' grapevine suspension cells or tobacco leaves.

	Res concentration in grape '41B' suspension cells (μg/g FW)		Res concentration in tobacco leaves (μg/g FW)	
	The control (DMSO)	The treatment (exogenous Res)	The control (DMSO)	The treatment (exogenous Res)
trans-Pd	0.00	15.61±0.49**	0.00	0.57±0.01**
cis-Pd	0.00	0.95±0.58	0.00	0.00
trans-Res	0.13±0.00	256.59±4.89**	0.00	29.27±1.09**
cis-Res	0.00	2.24±0.10	0.00	0.00
Total Res	0.13±0.00	275.39±5.66**	0.00	29.85±1.08**

Student's t-test was used to analyze significant differences, asterisks denote significant differences compared with the control, *, P <0.05; **, P < 0.01.

Table S4 The interacting proteins of VvWRKY8 by screening yeast two-hybrid library.

**Serial number**	**Gene ID**	**Gene position**	**Gene name**
1	GSVIVT01015952001	chr9:16094219..16096253	probable WRKY transcription factor 40-like [*Vitis vinifera*]
2	GSVIVT01028725001	chr16:19388139..19394697	PREDICTED:Ubiquitin-associated/translation elongation factor EF1B protein[*Vitis vinifera*]
3	GSVIVT01024167001	chr3:644718..647153	Arginine decarboxylase [*Vitis vinifera*]
4	GSVIVT01033674001	chr8:18768420..18771398	PREDICTED: E3 ubiquitin-protein ligase MIEL1 [*Vitis vinifera*]
5	GSVIVT01007647001	chr17:10869022..10874179	PREDICTED: protein NBR1 homolog isoform X2 [*Vitis vinifera*]
6	GSVIVT01027002001	chr15:18650540..18655050	PREDICTED: probable E3 ubiquitin-protein ligase RHB1A isoform X1 [*Vitis vinifera*]
7	GSVIVT01027994001	chr7:3060928..3067386	PREDICTED: universal stress protein PHOS34 [*Vitis vinifera*]
8	GSVIVT01020783001	chr12:2044341..2046532	PREDICTED:ACD32.1 encodes an alpha-crystallin domain containing protein with homology to small heat shock proteins. [*Vitis vinifera*]
9	GSVIVT01028328001	chr7:6210900..6212796	R2R3 MYB14 transcription factor [*Vitis vinifera*]
10	GSVIVT01022566001	chr8:5156853..5163974	PREDICTED: putative phosphatidylglycerol/phosphatidylinositol transfer protein DDB_G0282179 [*Vitis vinifera*]

11	GSVIVT01037696001	chr19:7005369..7014427	PREDICTED: cell differentiation protein RCD1 homolog isoform X2 [*Vitis vinifera*]
12	GSVIVT01025384001	chr6:1199805..1206412	PREDICTED: protein EMBRYO SAC DEVELOPMENT ARREST 3, chloroplastic [*Vitis vinifera*]
13	GSVIVT01031753001	chr3:4060722..4066802	PREDICTED: cell number regulator 1 isoform X1 [*Vitis vinifera*]
14	GSVIVT01034093001	chr8:15193127..15196432	PREDICTED: diacylglycerol kinase theta-like [*Vitis vinifera*]
15	GSVIVT01023827001	chr3:3165259..3173838	PREDICTED: COP9 signalosome complex subunit 5b [*Vitis vinifera*]
16	GSVIVT01021072001	chr5:14128823..14143945	PREDICTED: protein PHR1-LIKE 2 isoform X2 [*Vitis vinifera*]
17	GSVIVT01025674001	chr8:13029245..13030391	PREDICTED: probable E3 ubiquitin-protein ligase LOG2 [*Vitis vinifera*]
